# Activation of Dopamine D1-D2 Receptor Complex Attenuates Cocaine Reward and Reinstatement of Cocaine-Seeking through Inhibition of DARPP-32, ERK, and ΔFosB

**DOI:** 10.3389/fphar.2017.00924

**Published:** 2018-01-04

**Authors:** Ahmed Hasbi, Melissa L. Perreault, Maurice Y. F. Shen, Theresa Fan, Tuan Nguyen, Mohammed Alijaniaram, Tomek J. Banasikowski, Anthony A. Grace, Brian F. O'Dowd, Paul J. Fletcher, Susan R. George

**Affiliations:** ^1^Department of Pharmacology, University of Toronto, Toronto, ON, Canada; ^2^Departments of Neuroscience, Psychiatry and Psychology, University of Pittsburgh, Pittsburgh, PA, United States; ^3^Centre for Addiction and Mental Health, Toronto, ON, Canada; ^4^Departments of Psychology and Psychiatry, University of Toronto, Toronto, ON, Canada; ^5^Department of Medicine, University of Toronto, Toronto, ON, Canada

**Keywords:** addiction and addiction behaviors, dopamine D1-D2 heteromer, DARPP-32, deltaFosB, dopamine signaling, proximity ligation assay, FRET, addiction treatment

## Abstract

A significant subpopulation of neurons in rat nucleus accumbens (NAc) coexpress dopamine D1 and D2 receptors, which can form a D1-D2 receptor complex, but their relevance in addiction is not known. The existence of the D1-D2 heteromer in the striatum of rat and monkey was established using *in situ* PLA, *in situ* FRET and co-immunoprecipitation. In rat, D1-D2 receptor heteromer activation led to place aversion and abolished cocaine CPP and locomotor sensitization, cocaine intravenous self-administration and reinstatement of cocaine seeking, as well as inhibited sucrose preference and abolished the motivation to seek palatable food. Selective disruption of this heteromer by a specific interfering peptide induced reward-like effects and enhanced the above cocaine-induced effects, including at a subthreshold dose of cocaine. The D1-D2 heteromer activated Cdk5/Thr75-DARPP-32 and attenuated cocaine-induced pERK and ΔFosB accumulation, together with inhibition of cocaine-enhanced local field potentials in NAc, blocking thus the signaling pathway activated by cocaine: D1R/cAMP/PKA/Thr34-DARPP-32/pERK with ΔFosB accumulation. In conclusion, our results show that the D1-D2 heteromer exerted tonic inhibitory control of basal natural and cocaine reward, and therefore initiates a fundamental physiologic function that limits the liability to develop cocaine addiction.

## Introduction

There is significant evidence for the involvement of mesolimbic and striatal dopamine (DA) transmission in mediating different aspects of reward, as well as aversion, with particular importance for the nucleus accumbens (NAc) (reviewed in Salamone and Correa, 2012). Analysis of DA involvement through its receptors (Beaulieu and Gainetdinov, 2011) has focused on the mechanisms occurring in DA D1 receptor (D1R)-expressing or D2 receptor (D2R)-expressing medium spiny neurons (MSNs) in the striatum composing the two distinct projection pathways, the striatonigral D1R-enriched pathway and the striatopallidal D2R-enriched pathway. There is, however, a significant subpopulation of neurons that coexpress both D1R and D2R (Meador-Woodruff et al., 1991; Deng et al., 2006; Gangarossa et al., 2013; Gagnon et al., 2017) to form a D1-D2 receptor complex in the NAc (Hasbi et al., 2009; Perreault et al., 2010, 2016; Rico et al., 2017). The involvement of these MSNs and specifically the role of the D1-D2 heteromer complex activation in the modulation of brain reward functions and addiction mechanisms have not been studied.

The existence of such MSNs coexpressing D1R and D2R have been established by different methodologies including *in situ* hybridization of mRNAs (Meador-Woodruff et al., 1991; Weiner et al., 1991; Lester et al., 1993), single cell RT-PCR (Surmeier et al., 1996), double immunofluorescence or retrograde labeling methods (Shetreat et al., 1996; Wong et al., 1999; Deng et al., 2006). The colocalization in rat striatum was estimated by confocal double-immunofluorescence to occur in 20–25% of D1R-expressing MSNs in NAc and 6% of such neurons in caudate putamen (CPu) (Hasbi et al., 2009; Perreault et al., 2010). These results were also confirmed by estimations from BAC transgenic mice (Lee et al., 2006; Bertran-Gonzalez et al., 2008; Matamales et al., 2009), with a higher degree of D1R and D2R colocalization in ventral striatum (10–17%) than in dorsal striatum (1–6%), with up to 38% in the bundle-shaped subregion of the mouse caudomedial NAc shell (Gangarossa et al., 2013). Furthermore, neurons that coexpressed D1R and D2R in rat NAc showed a unique phenotype since they coexpressed enkephalin and dynorphin (Perreault et al., 2010) and also coexpressed GABA and glutamate (Perreault et al., 2012), in contrast to the two classical phenotypes of MSNs, D1R with dynorphin and D2R with enkephalin, respectively (Chesselet and Graybiel, 1983; Beckstead and Kersey, 1985). Physical interaction and heteromer formation between D1R and D2R in rat and human striatum was established by co-immunoprecipitation (Lee et al., 2004; Rashid et al., 2007; Hasbi et al., 2009, 2014; Pei et al., 2010), quantitative confocal FRET methodologies in rat NAc (Hasbi et al., 2009; Perreault et al., 2010) and GST pulldown in human striatum (Pei et al., 2010). The neurons expressing dopamine D1-D2 receptor heteromer in the NAc were able to influence neurotransmission within the ventral tegmental area (VTA) and substantia nigra (Perreault et al., 2012).

At present, there is no selective agonist for the D1-D2 receptor heteromer and the only known pharmacological tool, besides dopamine, capable of potently activating this receptor complex with high affinity leading to calcium mobilization was shown to be the D1-like ligand SKF 83959 (Rashid et al., 2007; Hasbi et al., 2009, 2014; Perreault et al., 2010, 2012). However, SKF 83959 can also bind with high affinities to D1 and D5 receptors and with much lower affinities to the other dopamine receptor subtypes (D2R, D3R, and D4R), and to other unrelated receptors, such as adrenoceptors, serotonin receptors and sigma-1 receptors (Andringa et al., 1999; Chun et al., 2013; Guo et al., 2013). While SKF 83959-induced calcium release in the striatum is highly likely due to the activation of the D1-D2 heteromer since the expression of D5R in this region is very low (Hasbi and George, 2010) and the calcium signal is blocked by either D1 or D2 antagonists (Rashid et al., 2007; Hasbi et al., 2009, 2014; Perreault et al., 2010), the selectivity of SKF 83959 toward the D1-D2 heteromer would be compromised in other brain regions (Perreault et al., 2012) or when G_q_ is highly expressed (Chun et al., 2013). Also, although the D1R or D2R antagonists we have tested blocked the D1-D2 heteromer-activated calcium signal (Lee et al., 2004; Rashid et al., 2007; Hasbi et al., 2009, 2014; Perreault et al., 2010), they would also block the individual D1R and D2R homomers *in vivo*. For these reasons, we designed a selective antagonist for the D1-D2 heteromer (Hasbi et al., 2014). This D1-D2 heteromer antagonist, the TAT-D1 peptide, is used to confirm or deny the involvement of the D1-D2 heteromer in the observed effects whenever SKF 83959 is used. This functional antagonist was devised to target the major site of interaction between D1R and D2R responsible for heteromer formation (O'Dowd et al., 2012). A pharmacological tool consisting of a small peptide was then generated from the D1R interaction site, capable of disrupting the physical interaction between D1R and D2R, resulting in the inhibition of D1-D2 receptor heteromer formation and its activated calcium signal (Hasbi et al., 2014). The effects of this D1-D2 heteromer disrupting peptide were shown to be highly selective *in vivo* and *in vitro* without effects on other homomers or heteromers such as D1-D1, D2-D2, D5-D5, D2-D5 (Hasbi et al., 2014), and D1-D3, D2-5HT_2A_ (present manuscript), and has helped to reveal important roles of the D1-D2 heteromer in depressive-like (Hasbi et al., 2014; Shen et al., 2015) and anxiety-like behavior (Shen et al., 2015) in animal models. We used this peptide in the present study to validate the involvement of the D1-D2 heteromer in brain reward function and mechanisms related to cocaine addiction, which evolves along stages including initiation, development, maintenance and relapse to cocaine abuse (Koob and Volkow, 2010).

DARPP-32 (dopamine- and cAMP-regulated phosphoprotein of MW 32 kDa) (Bibb et al., 1999; Greengard et al., 1999; Nairn et al., 2004; Svenningsson et al., 2004), extracellular regulated kinase (ERK) (Valjent et al., 2000, 2006; Bertran-Gonzalez et al., 2008), and ΔFosB (Nestler, 2005, 2008), are among the most important striatal proteins implicated in mediating the progression to cocaine addiction. Mechanistically, cocaine mediates its effects in part by blocking dopamine reuptake leading to elevated synaptic dopamine, which was shown to regulate the state of phosphorylation of DARPP-32 in a bidirectional manner. Activation of D1R, via stimulation of PKA, results in DARPP-32 phosphorylation at Thr34, rendering DARPP-32 a powerful inhibitor of protein phosphatase-1 (PP1). DARPP-32 has also been shown to be phosphorylated at Thr75 by a cyclin-dependent kinase (Cdk5) mechanism, which converts DARPP-32 into an inhibitor of the PKA pathway (Bibb et al., 1999; Nishi et al., 2000). DARPP-32 can therefore functionally act in an opposite manner as either an inhibitor of PP-1 or an inhibitor of the PKA pathway (Bibb et al., 1999; Greengard et al., 1999; Nairn et al., 2004; Svenningsson et al., 2004, 2005). Acute treatment with cocaine increases phosphorylation of Thr34-DARPP-32 and decreases phosphorylation at Thr75, whereas, chronic treatment (5 daily consecutive injections) increases phosphorylation of Thr75 (Bibb et al., 2001). Several well-known cocaine-induced behavioral effects were shown to depend on DARPP-32 activity: cocaine-induced CPP and cocaine self-administration are attenuated in DARPP-32 KO mice (Fienberg and Greengard, 2000; reviewed in Svenningsson et al., 2005), whereas, cocaine-induced development of locomotor sensitization was potentiated by intra-accumbal injection of Cdk5 inhibitors suggesting the involvement of Cdk5/Thr75-DARPP-32 in inhibiting sensitization (reviewed in Fienberg and Greengard, 2000; Nairn et al., 2004; 2005).

Cocaine induces activation (phosphorylation) of ERK in NAc MSNs through mechanisms involving the DARPP-32/PP1 cascade (Valjent et al., 2000, 2006; Svenningsson et al., 2004, 2005). Activation of ERK results in the direct or indirect phosphorylation of various transcription factors and leads to the induction of immediate-early genes that are essential for long-lasting behavioral alterations. One transcription factor, ΔFosB, has high importance in mediating the enduring effects of cocaine and other abused drugs in the NAc (McClung and Nestler, 2003; Nestler, 2008). This splice product of the *fosB* gene, accumulates in NAc only after repeated drug exposure (McClung and Nestler, 2003; Lobo et al., 2010; Lobo and Nestler, 2011), and has been hypothesized to be a potential molecular switch in the transition from recreational drug use to the chronically addicted state (Nestler, 2005, 2008). Cocaine-induced ΔFosB accumulation was observed in D1R MSNs within the NAc (Lobo et al., 2010), in line with data from ΔFosB-overexpression studies (Kelz et al., 1999; Nestler, 2005, 2008; Zachariou et al., 2006).

Since the D1-D2 receptor heteromer is principally localized in the NAc, the enhanced dopamine release following cocaine would also activate these D1-D2 receptor complexes in the NAc. We therefore investigated the effects of activating or specifically disrupting the D1-D2 receptor heteromer on different behavioral outputs involving reward mediated by cocaine. We also investigated the signaling pathway that may be involved in the D1-D2 heteromer-mediated effects, with a focus on the involvement of the major proteins involved in addiction, DARPP-32 ERK1/2 and ΔFosB.

## Results

### Evidence for the D1-D2 heteromer in rat and monkey striatum

**Figure 1:**
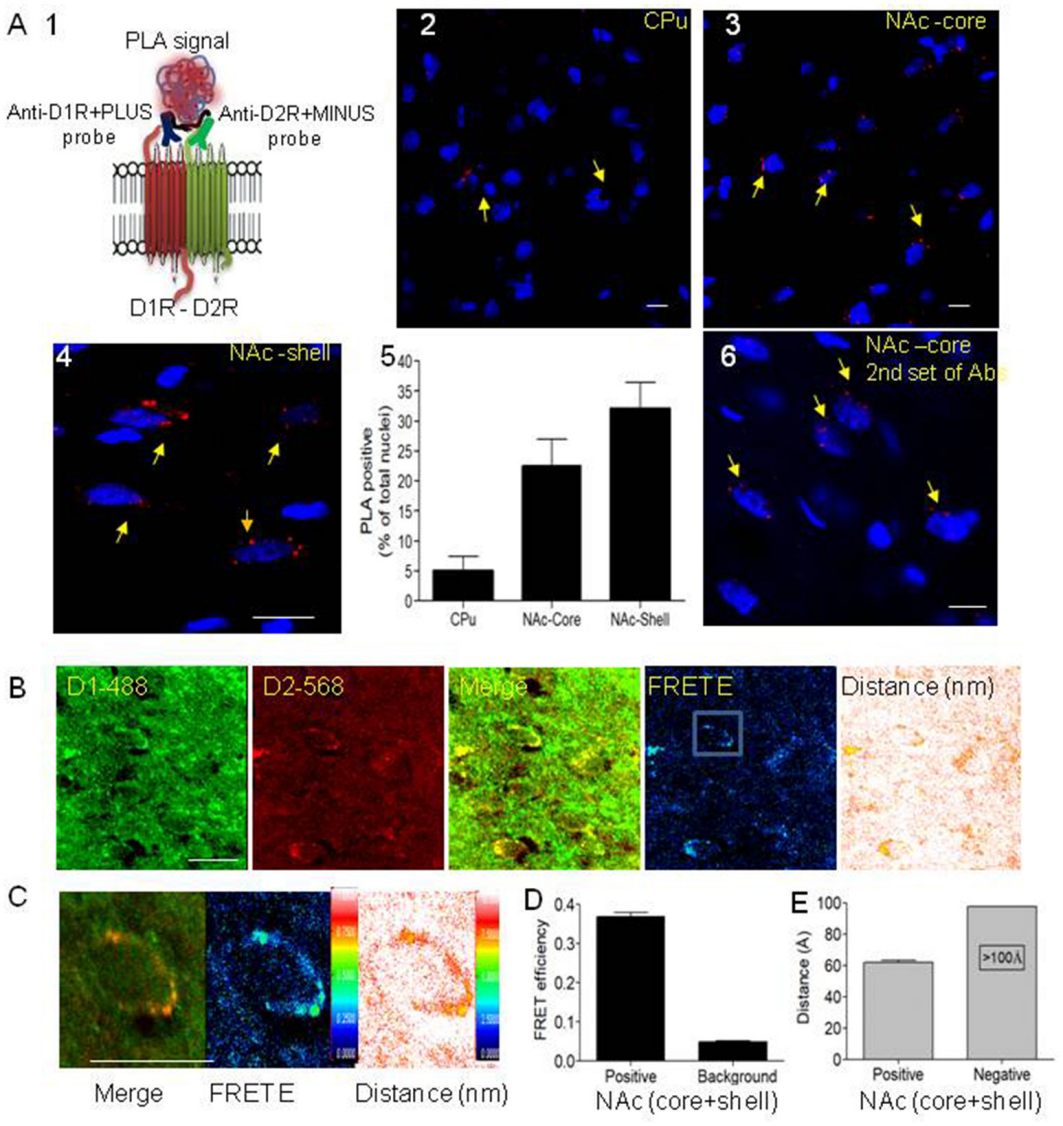
Evidence for the existence of dopamine D1-D2 receptor heteromer in rat NAc. **(A)** Proximity ligation assay (PLA) was used to visualize and detect D1R and D2R close proximity. **(A1)** A scheme depicts the PLA probes used in the present study. **(A2–A4)** Representative images of PLA signals (red dots) in neurons (nuclei stained by DAPI) in rat caudate putamen (CPu), nucleus accumbens core (NAc-core) and shell (NAc-shell) subregions. **(A5)** Graph representing the percent of neurons with a positive PLA signal. **(A6)** Representative image of PLA signals in neurons (nuclei stained by DAPI) in rat NAc-core using the second set of antibodies. **(B)** Representative images of immunohistochemistry using D1R antibody (D1R-Ab) or D2R antibody (D2R-Ab) directly conjugated to Alexa-488 or Alexa-568, respectively, in the NAc-shell. Direct confocal FRET analysis was performed, reflected by FRET efficiency (FRET E) and the distance between the dipoles, less than 10 nm (100 Å). **(C)** A representative close-up of a single MSN cell body from NAc showing D1R-D2R colocalization (left), D1-D2 heteromer FRET efficiency (center) and relative distance between receptors (right). **(D,E)** Histograms showing FRET E ratios **(D)** and distance **(E)** obtained from MSN cell bodies from NAc (*n* = 24). Bars are 10 μm.

To provide evidence and direct visualization of D1-D2 heteromers in rat striatum, we performed the *in situ* proximity ligation assay (*in situ* PLA) and *in situ* confocal FRET. The PLA technique was previously used to show receptor-receptor interaction between different G-protein coupled receptors (GPCRs) (Borroto-Escuela et al., 2013, 2016). Since the existence of D1-D2 heteromers was challenged based notably on the failure to observe a PLA signal in mouse striatum (Frederick et al., 2015), we used the same two sets of antibodies used in that study. The PLA signal can only be generated when the two PLA probes have bound in close proximity; we directly conjugated the two sets of antibodies to oligonucleotides to generate the PLA probes, thus avoiding the use of secondary antibodies (Figure 1A1). The PLA performed with the well-validated primary antibodies (Lee et al., 2004; Perreault et al., 2010, 2016; present study) to D1R (Rat, Sigma D2944) and D2R (Rabbit, Millipore AB5084) showed PLA signals visualized as clear fluorescent red signals at the cell body, suggesting a close proximity of D1R and D2R in MSNs of rat CPu, NAc-core and NAc-shell (Figures 1A2–A4). Analysis of these images using a PLA-dedicated Duolink software revealed that while in the CPu the number of neurons with PLA signal was low (5% ± 2.4 [437 neurons]), D1-D2 heteromers were present in more than 22% of MSNs (22.6% ± 4.2 [604 neurons]) in the NAc-core and more than 30% of MSNs (32 ± 4.1 [732 neurons]) in rat NAc-shell (Figure 1A5). Controls for PLA and Z-stack images were performed to validate the signal and its cell surface localization (Supplementary Figures 1–4 and Supplementary Video 1). Thus, no PLA signal was observed in the absence of either of the two probes, the ligase or the polymerase (Supplementary Figure 1A). The PLA signal was observed in the dorsal and ventral striatum of D5 KO mouse, whereas it was absent in D1 KO and D2 KO mouse striatum, a clear indication of the specificity of the probes (Supplementary Figure 1B). Pretreatment with the TAT-D1 peptide (300 pmol, i.c.v.), and not the TAT-scrambled control peptide, abolished the D1-D2 PLA signal (Supplementary Figure 2) demonstrating the specificity of the PLA signal and clearly indicating that the PLA signal came from D1-D2 heteromers. Z-stacks (15–20 series, 0.9–1.7 μm) were taken and their analysis showed that the PLA signal was around nuclei, suggesting that D1-D2 heteromers were at the cell surface around the cell bodies (Supplementary Figures 3, Supplementary Video 1). No nuclear PLA signal was observed in line with the non-existence of D1R and D2R in cell nucleus. Moreover, using a second set of antibodies which consisted of the same anti-D1R and an antibody generated against D2R by that group (Frederick et al., 2015) and commercialized by Millipore (ABN 462), we also detected a clear PLA signal in rat NAc (Figure 1A6) indicating the presence of D1-D2 heteromers. Furthermore, we also detected a PLA signal in monkey NAc and caudate, indicative of a close proximity between dopamine D1 and D2 receptors in non-human primate as well (Supplementary Figure 4), and which was absent in negative controls. These results are in agreement with another study in macaques showing PLA between D1 and D2 receptors (Rico et al., 2017). In contrast to the high nuclear labeling in the above mentioned study (Frederick et al., 2015), we did not observe any non-specific nuclear labeling in any of our PLA studies.

Since PLA is an indication of colocalization and protein-protein interaction (in a radius of 0–40 nm), we also used IHC and direct *in situ* confocal FRET, in which the primary antibodies for D1R (Rat, Sigma, D2944) and D2R (Millipore AB5084P) (Lee et al., 2004; Perreault et al., 2010, 2016) were directly conjugated to fluorophores (Alexa-488 and Alexa-568, respectively), thus avoiding the use of secondary antibodies. The IHC (Figure 1B and Supplementary Figure 5) followed by FRET shows positive FRET only when the two probes are in a very close proximity of less than 10 nm (<100 Å). FRET analysis showed robust FRET efficiency of 0.36 ± 0.03 in the NAc (core and shell), with a relative distance of 6 nm (60 Å) between the probes (Figures 1C–E). These results indicate clearly that the two receptors are not only colocalized but at a receptor-receptor distance compatible with direct physical interaction with each other. In the CPu, there was no significant FRET in the few neurons where colocalization was observed. These results from PLA and direct FRET are clear confirmation of the existence of the D1-D2 heteromer in a subset of MSNs in rat NAc core and shell, and which is scarce if not absent in the MSNs of the dorsal striatum.

### Effects of the activation and inactivation of the D1-D2 receptor heteromer on reward related behavior

The stages of development of addiction are mirrored in behavioral tests used to study different aspects: conditioned place preference or aversion (CPP/CPA), drug self-administration (SA) and locomotor sensitization. CPP/CPA tests behavior to seek drug reward or its avoidance. Repeated injections of psychoactive drugs lead to locomotor sensitization. Finally, the SA paradigm models voluntary drug taking and seeking. After abstinence, reinstatement to SA models a relapse to drug use.

#### Effects of the disruption and activation of the D1-D2 receptor heteromer on basal CPP

**Figure 2:**
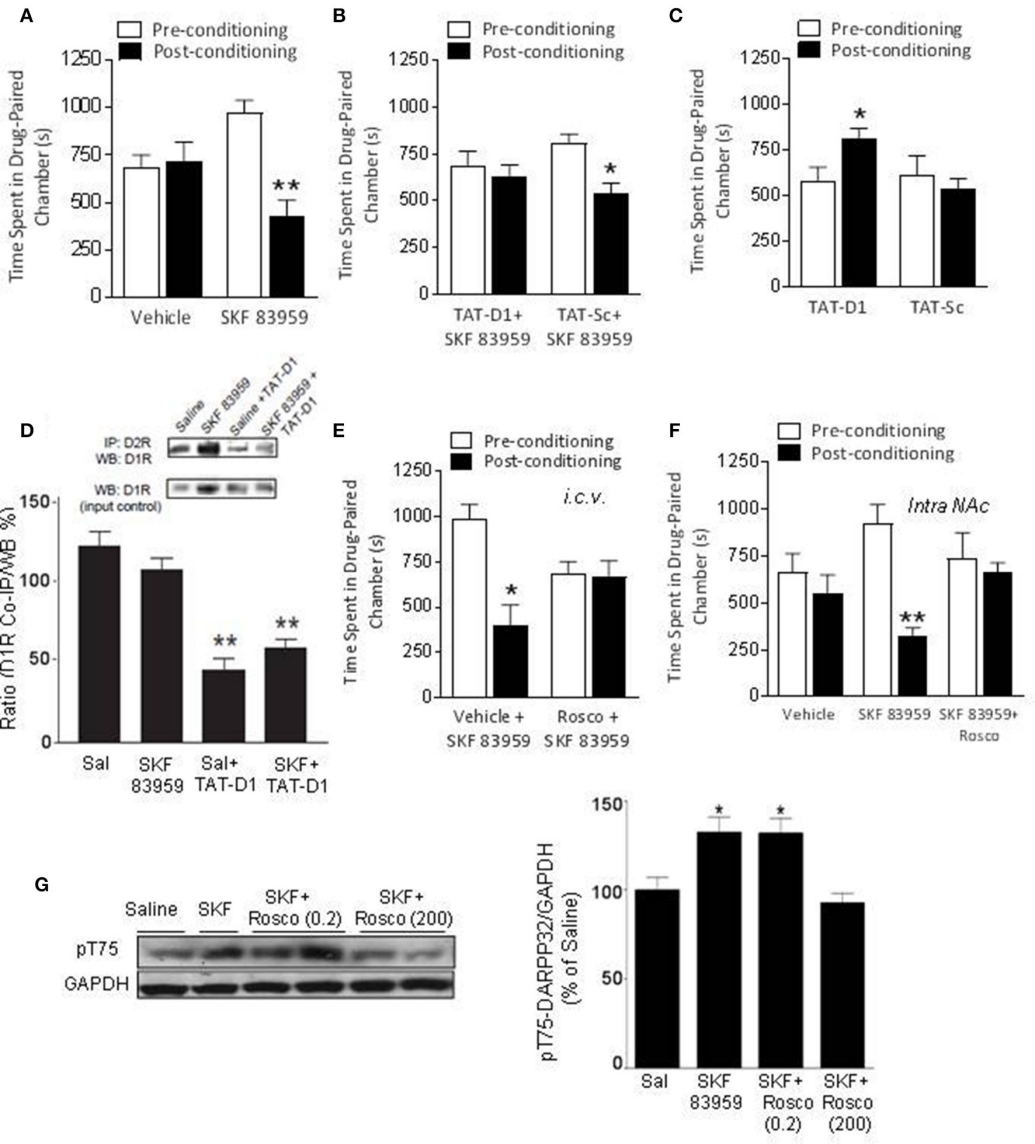
The effects of D1-D2 heteromer stimulation and inactivation on basal conditioned place preference. **(A)** Vehicle-conditioned rats did not exhibit a preference toward a particular chamber. D1-D2 heteromer stimulation by SKF 83959 (1.5 mg/kg, s.c.) induced conditioned place aversion (CPA) as the animals spent significantly less time in the drug paired chamber. **(B)** SKF 83959-induced CPA was abolished by pre-treatment by the D1-D2 heteromer selective disrupting peptide, TAT-D1, but not the control TAT-Sc peptide. **(C)** Inactivation of D1-D2 heteromer by TAT-D1 resulted in conditioned place preference (CPP) as the rats spent significantly more time in the drug paired chamber, not observed with the control TAT-Sc. **(D)** Representative western blots (inset) and histogram showing the amount of D1R co-immunoprecipitated with D2R from the NAc of rats treated with saline or SKF 83959. Pretreatment with TAT-D1 led to decreased co-immunoprecipitated receptors. An aliquot of each sample was used as a control for WB (input control). **(E,F)** The CPA induced by D1-D2 heteromer stimulation was abolished by Cdk5 inhibitor roscovitine pre-treatment (200 nmol, i.c.v, **E**) or intra-accumbal injection (30 nmol, **F**). **(G)** Representative western blot and histogram showing the density of Thr75-DARPP-32 phosphorylation (pT75) relative to GAPDH (as loading control). Data represent means ± SEM of *n* = 8–10 rats/group. (^*^p < 0.05, ^**^p < 0.01: compared to saline).

After habituation during which the preference of each rat was noted (pre-conditioning preference), rats received either saline or SKF 83959 (1 mg/kg, s.c, 6 alternate sessions: D-S-D-S-D-S) during the conditioning phase and their preference for the drug-paired environment on the test day was compared to their preconditioning preference for the same chamber [Figures 2A,B, ANOVA, *F*_(3, 29)_ = 4.21; *p* = 0.014; Table 1]. Control animals received saline in both chambers and did not exhibit preference toward a specific chamber on average (Figure 2A). Rats that received SKF 83959 during the conditioning phase spent significantly less time in the SKF 83959-paired compartment post-conditioning compared to pre-conditioning (baseline) [Figure 2A, SKF: *t*_(8)_ = 3.72, *p* = 0.006] indicative of conditioned place aversion (CPA). The involvement of the D1-D2 receptor heteromer in the SKF 83959-induced place aversion was confirmed by the use of the selective heteromer disrupting TAT-D1 peptide, which has no effect on other homomers and heteromers (Hasbi et al., 2014) including D1-D3 and D2-5HT_2A_ (Supplementary Figure 6). The animals were treated with SKF 83959 15 min after either the TAT-D1 peptide or the TAT-scrambled peptide (300 pmol, i.c.v.) during the conditioning sessions. SKF 83959-induced CPA was abolished by pretreatment with the TAT-D1 peptide but not by the scrambled peptide (TAT-sc) [Figure 2B, TAT-D1: *t*_(7)_ = 0.38; *p* = 0.0.718; TAT-sc: *t*_(7)_ = 2.80; *p* = 0.026], clearly indicating that the CPA resulting from SKF 83959 treatment was mediated through the D1-D2 heteromer. Moreover, and in contrast to the CPA observed by the SKF 83959-induced activation of the D1-D2 heteromer, disruption of the basal concentration of D1-D2 receptor heteromer by the TAT-D1 peptide alone, resulted in place preference (CPP), not seen with the scrambled peptide [Figure 2C, ANOVA, *F*_(2, 28)_ = 11.07; *p* = 0.000; Veh: *t*_(12)_ = −1.30, *p* = 0.550; TAT-D1: *t*_(8)_ = −0.87; *p* = 0.005; TAT-sc: *t*_(5)_ = 1.21; *p* = 0.281]. The effects of TAT-D1 on the heteromer in NAc of these animals were also analyzed using co-immuprecipitation experiments (Figure 2D). The results showed that D1R was co-immunoprecipitated with D2R from the NAc of rats injected with saline or SKF 83959. Treatment with TAT-D1 led to more than 50% decrease in the amount of D1R co-immunoprecipitated with D2R in saline- as well as in SKF 83959-injected animals, suggesting that TAT-D1 decreased the amount of D1-D2 heteromer in the NAc of these animals, leading to the abolishment of SKF 83959-induced CPA.

**Table 1:** Statistical analyses for the individual experiments.

**Figure**	**Groups, *N***	**Test**	***Post-hoc***		
Figures 2A,B	Vehicle, 8; SKF 83959, 9;SKF+TAT-D1, 8; SKF+TAT-sc, 8	ANOVA; *F*_(3, 29)_ = 4.21; *p* = 0.014	SKF83959: *t*_(8)_ = 3.72; *p* = 0.006		
			SKF+TAT-D1: *t*_(7)_ = 0.38; *p* = 0.718		
			SKF+TAT-sc: *t*_(7)_ = 2.80; *p* = 0.026		
Figure 2C	Vehicle,13;TAT-D1, 9; TAT-sc, 6	ANOVA; *F*_(2, 28)_ = 11.07; *p* = 0.000	Veh: *t*_(12)_ = −1.30, *p* = 0.550		
			TAT-D1: *t*_(8)_ = −0.87; *p* = 0.005		
			TAT-sc: *t*_(5)_ = 1.21; *p* = 0.281		
Figures 2D,E	Vehicle+SKF, 6; Roscovitine+SKF, 6	Two-tailed *t*-test; *t*_(10)_ = 2.27	Veh+SKF: *t*_(5)_ = 2.74; *p* = 0.041		
			Rosco+SKF: *t*_(5)_ = 0.13; *p* = 854		
Figure 2G WB-Roscovitine Effect		ANOVA, *F*_(3, 30.59)_; *p* < 0.0001	Sal vs. SKF: *t* = 5.908, *p* < 0.001		
		*Post-hoc*: Bonferoni's multiple comparisons	Sal vs. SKF+Rosco (0.2): *t* = 5.485; *p* < 0.001		
			Sal vs. SKF+Rosco (200): *t* = 1.075; *p* > 0.05		
			SKF vs. SKF+Rosco (0.2): *t* = 5.028; *p* > 0.05		
			SKF vs. SKF+Rosco (200): *t* = 7.596; *p* < 0.001		
			SKF+Rosco (0.2) vs. SKF+Rosco (200): *t* = 7.24; *p* < 0.001		
Figures 3C,D (IHC-pT75)	Saline 3 rats; SKF 83959 3 rats	Two tailed *t*-test	D1-Enk: *t*_(174)_ = 8.37; *p* < 0.0001		
	At least 3 slices from each rat Bregma: 1.6+/−0.6	Sal vs. SKF	Enk-only: *t*_(126)_ = 0.59; *p* = 0.55		
	Number of neurons are indicated in the figures		D1-only: *t*_(106)_ = 0.083; *p* = 0.93		
Figures 3E,F (IHC-pT34)	Saline 3 rats; SKF 83959 3 rats	Two tailed *t*-test	D1-Enk: *t*_(217)_ = 8.296; *p* < 0.0001		
	At least 3 slices from each rat Bregma: 1.6+/−0.6	Sal vs. SKF	Enk-only: *t*_(122)_ = 0.1.218; *p* = 0.2256		
	Number of neurons are indicated in the figures		D1-only: *t*_(120)_ = 0.1027; *p* = 0.9184		
Figures 3A,B (WB-pT75)	Vehicle 7; SKF 83959 7 rats	Two tailed *t*-test	pT75-15 min: *t*_(13)_ = 0.6164; *p* = 0.2738		
		Sal vs. SKF	pT75-45 min: *t*_(13)_ = 0.6885; *p* = 0.2512		
			pT75-90 min: *t*_(13)_ = 3.380; *p* = 0.0049		
Figures 3A,B (WB-pT34)	Vehicle 7; SKF 83959 7 rats	Two tailed *t*-test	pT34-15 min: *t*_(13)_ = 1.382; *p* = 0.0943		
		Sal vs. SKF	pT34-45 min: *t*_(13)_ = 0.2700; *p* = 0.3956		
			pT34-90 min: *t*_(13)_ = 0.5688; *p* = 0.2892		
Figure 4A	Coc, 9; Coc+SKF, 8; Coc+TAT-D1, 7	ANOVA; *F*_(2, 21)_ = 5.00; *p* = 0.017	Coc: *t*_(8)_ = −2.52; *p* = 0.036		
Acquisition CPP		*Post-hoc*: *t*-test	Coc+SKF: *t*_(7)_ = 0.97; *p* = 0.364		
			Coc+TAT-D1: *t*_(6)_ = −3.28; *p* = 0.017		
Figure 4B	Sal/SKF59, 8; Coc/Veh, 9; Coc/SKF59, 8; Coc/TAT-D1, 7	ANOVA; *F*_(3, 28)_ = 3.26; *p* = 0.036	Sal/Veh: *t*_(7)_ = −0.63; *p* = 0.550		
Expression CPP		*Post-hoc*: *t*-test	Sal/SKF59: *t*_(7)_ = −0.38; *p* = 0.713		
			Coc/Veh: *t*_(8)_ = −3.56; *p* = 0.007		
			Coc/SKF59: *t*_(8)_ = 0.49; *p* = 0.635		
Figure 4B		ANOVA; *F*_(3, 36)_ = 2.39; *p* = 0.086			
Figure 4C	7 to 9 rats each condition	Repeated measures ANOVA			
		Treatment effect: *F*_(5, 44)_ = 36.75, *p* < 0.0001			
		Treatment × injection: *F*_(30, 264)_ = 4.35, *p* < 0.0001			
		ANOVA Day 1: *F*_(5, 44)_ = 9.87; *P* < 0.0001			
		ANOVA Day 7: *F*_(5, 44)_ = 31.21; *P* < 0.0001			
Figure 4D		ANOVA treatment effect: *F*_(6, 52)_ = 18.83; *P* < 0.0001			
Figure 4E		ANOVA within subject effect: *F*_(3, 25)_ = 3.62; *P* = 0.030			
Figures 4F–H		ANOVA within subject effect: *F*_(5, 65)_ = 11.99; *P* < 0.0001			
Figures 4I,J		ANOVA within subject effect: *F*_(5, 65)_ = 11.99; *P* < 0.0001	*t*_(10)_ = 3.29; *p* < 0.01		
			*t*_(9)_ = 3.70; *p* < 0.01		
Figure 5A WB-pT75	Sal/Veh; Coc/Veh,; Coc/SKF; Sal/SKF	ANOVA	Bonferroni's multiple comparison test	t	Summary
		*F*_(3, 33)_ = 6.93, *p* = 0.001	Sal/Veh vs. SKF59/Veh	2.385	ns
			Sal/Veh vs. Coc/Veh	1.2	ns
			Sal/Veh vs. Coc/SKF59	4.35	^***^
			SKF59/Veh vs. Coc/Veh	1.263	ns
			SKF59/Veh vs. Coc/SKF59	1.684	ns
			Coc/Veh vs. Coc/SKF59	3.15	^*^
Figure 5A WB-pT34	Sal/Veh; Coc/Veh; Coc/SKF; Sal/SKF	ANOVA	Bonferroni's Multiple Comparison Test	t	
		*F*_(3, 33)_ = 1.098, *p* = 0.3652	Sal/Veh vs. SKF59/Veh	0.4261	ns
			Sal/Veh vs. Coc/Veh	0.3037	ns
			Sal/Veh vs. Coc/SKF59	1.215	ns
			SKF59/Veh vs. Coc/Veh	0.142	ns
			SKF59/Veh vs. Coc/SKF59	1.562	ns
			Coc/Veh vs. Coc/SKF59	1.518	ns
Figure 5B WB-pERK42	Sal/Veh; Coc/Veh; Coc/SKF; Sal/SKF	ANOVA	Bonferroni's multiple comparison test	t	Summary
		*F*_(3, 33)_ = 9.541	Sal/Veh vs. SKF59/Veh	0.3113	ns
			Sal/Veh vs. Coc/Veh	4.5	^***^
			Sal/Veh vs. Coc/SKF59	0.7415	ns
			SKF59/Veh vs. Coc/Veh	4.52	^***^
			SKF59/Veh vs. Coc/SKF59	1.005	ns
			Coc/Veh vs. Coc/SKF59	3.758	^**^
Figure 5B WB-pERK44	Sal/Veh; Coc/Veh; Coc/SKF; Sal/SKF	ANOVA	Bonferroni's Multiple Comparison Test	t	
		*F*_(3, 33)_ = 9.314	Sal/Veh vs. SKF59/Veh	0.6827	ns
			Sal/Veh vs. Coc/Veh	4.263	^**^
			Sal/Veh vs. Coc/SKF59	0.5302	ns
			SKF59/Veh vs. Coc/Veh	3.305	^*^
			SKF59/Veh vs. Coc/SKF59	1.179	ns
			Coc/Veh vs. Coc/SKF59	4.793	^***^
Figures 5C,D		Two tailed *t*-test	D1-Enk: *t*_(150)_ = 6.430; *p* < 0.0001		
IHC-pERK		Sal vs. SKF	D1-only: *t*_(131)_ = 0.12; *p* = 0.86		
Core			Enk-only: *t*_(80)_ = 0.165; *p* = 0.90		
Figures 5C,D		Two tailed *t*-test	D1-Enk: *t*_(148)_ = 6.691; *p* < 0.0001		
IHC-pERK		Sal vs. SKF	D1-only: *t*_(127)_ = 0.0609; *p* = 0.9515		
Shell			Enk-only: *t*_(77)_ = 1; *p* = 0.2399		

These results suggested a tonic inhibitory role of the D1-D2 heteromer on reward related behavior, which could be amplified by the activation and relieved by the disruption of the D1-D2 heteromer.

#### Involvement of DARPP-32 in D1-D2 heteromer-mediated CPA

DARPP-32 plays an important bidirectional role in DA transmission through its phosphorylation at Thr34 (pT34) or Thr75 (pT75) sites. Analysis of DARPP-32 phosphorylation in NAc by WB after SKF 83959 in preliminary experiments revealed no change in pT34 but an increase in pT75. We then examined the involvement of DARPP-32 phosphorylation at Thr75 by Cdk5 in the observed D1-D2 heteromer-mediated CPA. Complete inhibition of SKF 83959-induced CPA was obtained with injection of roscovitine (200 nmoles, i.c.v.), an inhibitor of Cdk5, 15 min before SKF 83959 (Figures 2E,F, Statistics below and Table 1), suggesting that Cdk5 activation may be responsible for the place aversion mediated by the D1-D2 receptor heteromer. Moreover, similar abolishment of SKF 83959-induced CPA was observed in rats injected with roscovitine (30 nmol/0.5μL) directly into the NAc bilaterally (Supplementary Figure 7A), in comparison to the CPA showed by rats that received vehicle 15 min before SKF 83959 [Figure 2F, *t*-test; *t*_(10)_ = 2.27; Veh+SKF: *t*_(5)_ = 2.74; *p* = 0.041; Rosco+SKF: *t*_(5)_ = 0.13; *p* = 854]. Western blot analysis using NAc homogenates from these animals showed that the phosphorylation of Thr75-DARPP32 increased by 33 ± 8% in the NAc of SKF 83959-treated rats in comparison with saline treatment [Figure 2G, ANOVA, *F*_(3, 31)_ = 30.59; *p* < 0.0001]. *Post-hoc* analysis using Bonferroni's Multiple Comparisons (Table 1) showed that pretreatment with a very low dose of roscovitine (0.2 nmoles, i.c.v.) had no significant effect on the SKF 83959-induced increase in pT75 (31.9 ± 8.3% over saline animals, SKF vs. SKF+Rosco (0.2): *t* = 5.028; *p* > 0.05). However, pretreatment with 200 nmoles roscovitine led to an abolishment of the SKF 83959-induced increase in pT75 (Figure 2G) [92.7 ± 5.33% of saline animal values, SKF vs. SKF+Rosco (200): *t* = 7.596; *p* < 0.001], in line with the blockade of the CPA at this dose (Figure 2E). These results indicated that conditioning with SKF 83959 stimulated Cdk5-mediated phosphorylation of Thr75-DARPP-32, which was essential for the CPA observed.

The modulation of DARPP-32 by the D1-D2 heteromer was investigated further by immunochemistry and western blot in rat striatum.

#### Modulation of DARPP-32 by D1-D2 receptor heteromer activation in rat striatum

**Figure 3:**
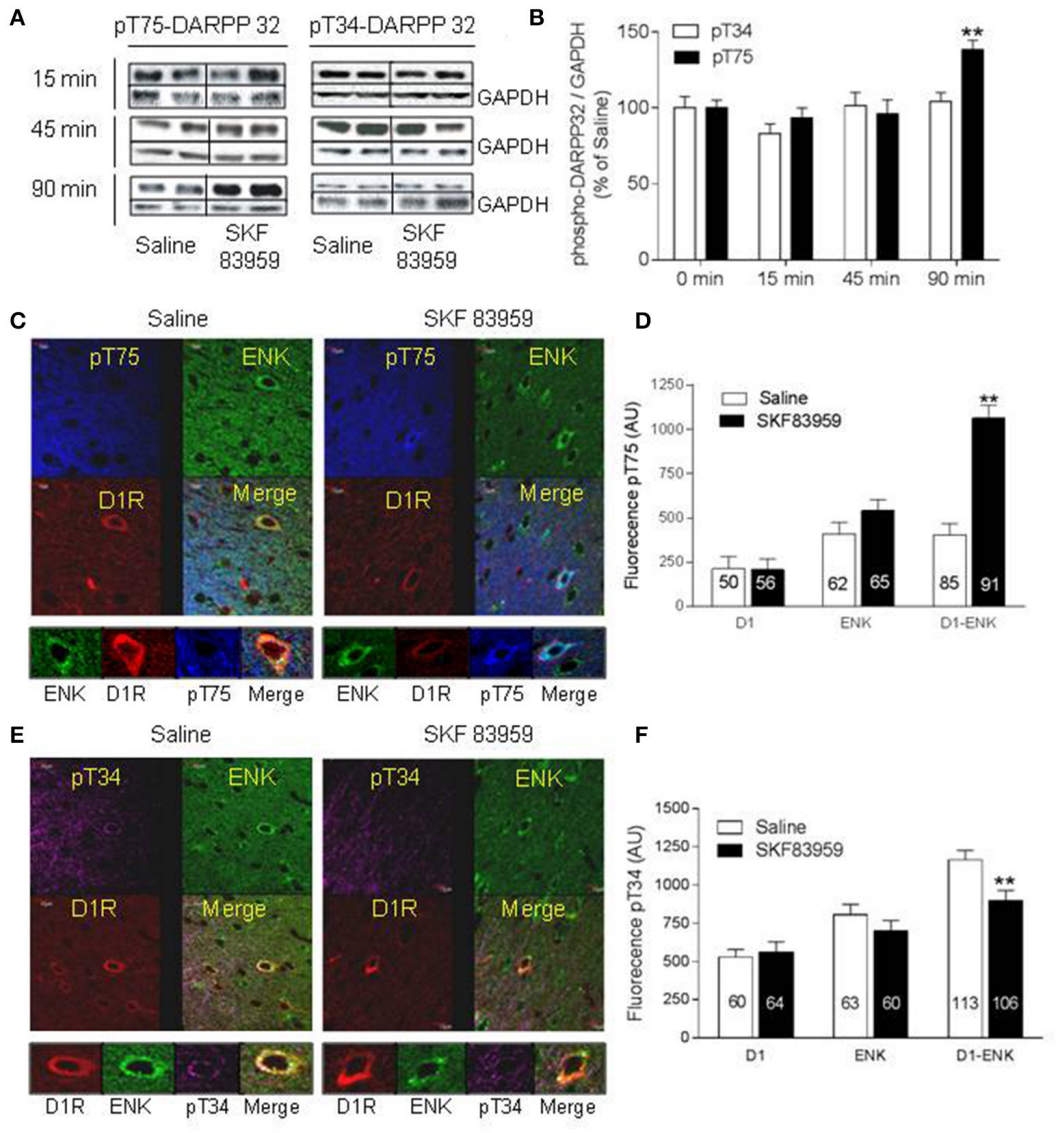
Activation of Thr75-DARRP-32 by D1-D2 heteromer in rat NAc. **(A,B)** Rats (*n* = 8/group) were injected with saline or SKF 83959 (1.5 mg/kg, s.c.), sacrificed 15, 45, or 90 min later, and phospho-Thr34-DARPP-32 (pT34) or phospho-Thr75-DARPP-32 (pT75) analyzed by western blot with GAPDH as loading control. **(A)** Representative blots of pT34 and pT75. **(B)** Quantification of blots from all animals represented as % mean ± SEM of control (saline values), (^**^p < 0.05). **(C–F)** Rats were injected with saline or SKF 83959 (1.5 mg/kg), sacrificed 15 min later, and immunohistochemistry performed using anti-pT34 or anti-pT75 assessed in the three types of MSNs: D1R-only (red arrow), Enk-only (D2R, green arrow) or D1R and ENK (D1-D2 heteromer)-expressing neurons (yellow arrow). **(C)** Representative confocal images of pT75-DARPP-32. **(D)** Quantification of pT75-DARPP-32 fluorescence in MSNs (*n* = numbers of neurons from at least *N* = 3 rats/condition). **(E)** Representative confocal images of pT34-DARPP-32. **(F)** Quantification of pT34 fluorescence in MSNs. Data represents means ± SEM after removal of non-specific background. (^**^p < 0.01).

Rats were injected with saline or SKF 83959 (1.5 mg/kg, s.c.), and sacrificed 15, 45, or 90 min later. Western blot analysis (Figures 3A,B) revealed a significant increase in pT75 (46 ± 8%) in NAc, only at 90 min after treatment with SKF 83959 compared to saline [*t*-test Sal vs. SKF, pT75-15 min: *t*_(13)_ = 0.6164; *p* = 0.2738; pT75-45 min: *t*_(13)_ = 0.6885; pT75-90 min: *t*_(13)_ = 3.380; *p* = 0.0049]. The pT34 level showed no change in NAc [Figures 3A,B, pT34-15 min: *t*_(13)_ = 1.382; *p* = 0.0943; pT34-45 min: *t*_(13)_ = 0.2700; *p* = 0.3956; pT34-90 min: *t*_(13)_ = 0.5688; *p* = 0.2892].

We then sought to investigate the cell specific changes in DARPP-32 induced by SKF 83959 using immunohistochemistry at earliest time points, in the three possible types of MSNs, those expressing D1R or D2R individually and those coexpressing both receptors. For this purpose, neurons from rat brain slices were immunolabeled for D1R and for enkephalin (Enk), which is a specific marker for D2R expressing neurons. We used ENK instead of D2R immunolabeling due to the fact that the most reliable antibodies for DARPP-32 and D2R were raised in the same host, rabbit. We have shown previously that any neuron coexpressing ENK and D1R was obligatorily expressing D2R also (Perreault et al., 2010). Cells positive for D1R and negative for ENK were considered as neurons expressing D1R only. Cells positive for ENK but negative for D1R labeling were designated as neurons expressing D2R only, whereas cells positive for both D1R and ENK (D1-Enk) labeling were designated as neurons expressing the D1-D2 receptor heteromer in the NAc. In CPu colocalization was rarely observed (<5% of D1R-MSNs) with very low heteromer formation (Hasbi et al., 2009; Perreault et al., 2010, and present results).

In comparison with saline-treated rats, the phosphorylation of Thr75-DARPP-32 in the NAc core and shell (Figures 3C,D) subregions of rats after treatment with SKF 83959 for 15 min showed a marked increase within neurons that coexpressed D1R and ENK [yellow arrow, *t*-test Sal vs. SKF, D1-Enk: *t*_(174)_ = 8.296; *p* < 0.0001], whereas no significant effect was observed in neurons that solely expressed D1R [red arrow, *t*_(105)_ = 0.083, *p* = 0.93] or ENK [D2R, green arrow, *t*_(126)_ = 0.59, *p* = 0.55] (Figures 3C,D). The SKF 83959-induced pT75 increase in D1-ENK neurons was inhibited in animals pretreated with TAT-D1 (300 pmol, i.c.v; Supplementary Figure 8, suggesting that the effect was D1-D2 heteromer mediated.

In rat NAc, 15 min after injection of SKF 83959, pT34 (Figures 3E,F) showed a significant decrease in D1-ENK neurons [i.e., expressing D1-D2 heteromer, yellow arrow, *t*_(217)_ = 8.296, *p* < 0.0001], whereas no significant effect was observed in neurons that expressed ENK [green arrow, *t*_(122)_ = 0.1.218; *p* = 0.2256] or D1R individually [D1-only: *t*_(120)_ = 0.1027; = 0.9184]. It was noted that the basal phosphorylation of pT34 was higher in the D1-ENK coexpressing neurons (yellow arrow) than in the neurons expressing D1R (red arrow) or ENK (green arrow) individually (Figure 3E). This effect was observed in both core and shell, with a prominent effect in the shell subregion.

These results suggested that activation of the D1-D2 receptor heteromer induced a dual modulation of DARPP-32, increasing phosphorylation at Thr75 and decreasing phosphorylation at Thr34 exclusively in D1-D2 expressing neurons in the NAc, and that the modulation of DARPP-32 by the D1-D2 heteromer was involved in the CPA observed.

### Effects of D1-D2 heteromer on cocaine-induced reward

#### Activation of D1-D2 receptor heteromer abolished and heteromer disruption enhanced cocaine-induced conditioned place preference (CPP)

**Figure 4:**
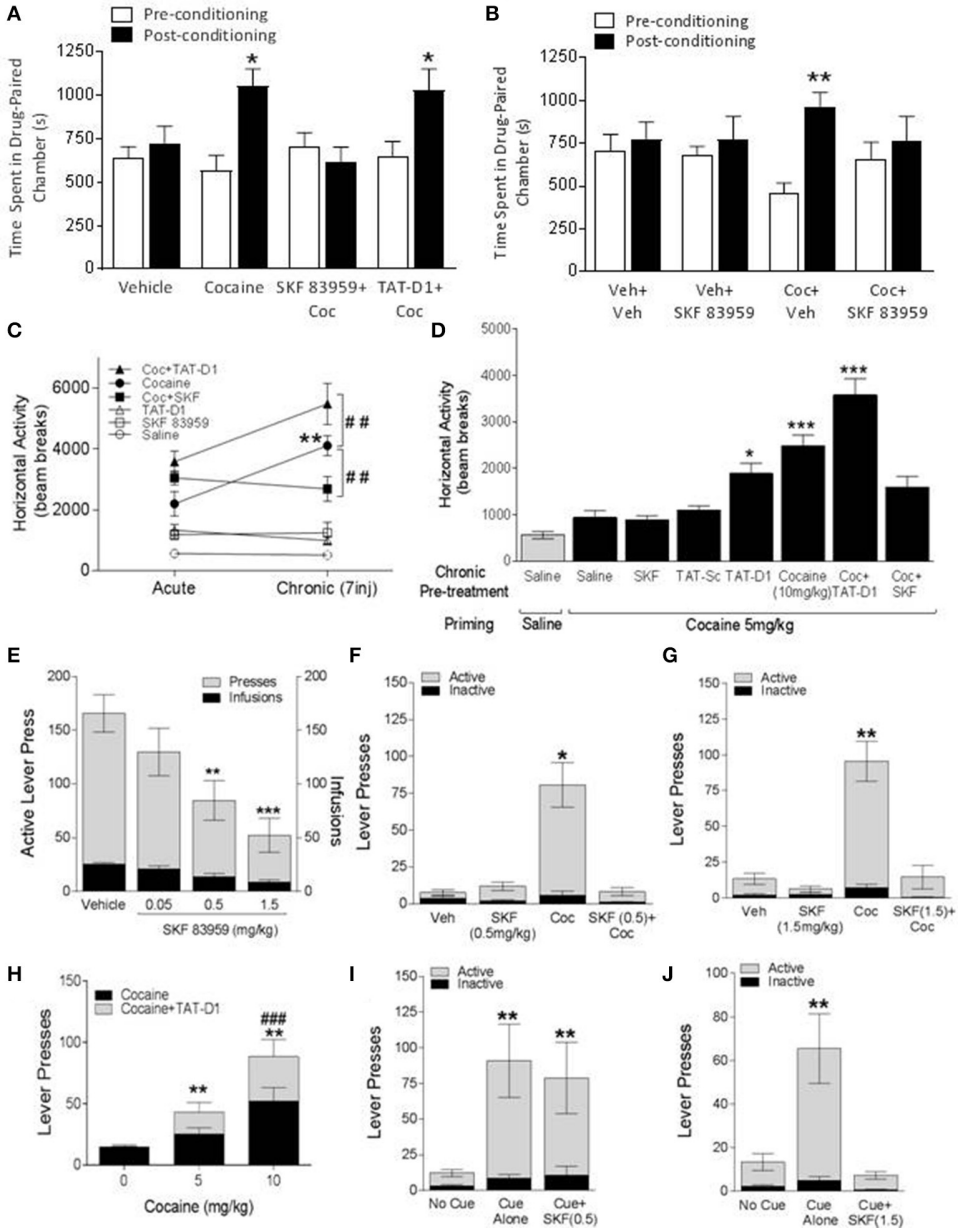
The effects of D1-D2 heteromer stimulation and inactivation on cocaine-induced behaviors. **(A,B)** Effects on cocaine CPP. **(A)** Vehicle-conditioned rats did not exhibit a preference toward a particular chamber. Cocaine-conditioned animals exhibited conditioned place preference (CPP). The acquisition of cocaine CPP was abolished by SKF 83959 and enhanced by TAT-D1 peptide (300 pmoles, i.c.v.). **(B)** A single injection of vehicle or SKF 83959 did not affect the chamber preference of vehicle-conditioned animals but abolished the expression of cocaine CPP in cocaine-conditioned animals. Data in **(A,B)** represent means ± SEM. of *n* = 8–10 rats/group. (^*^p < 0.05, ^**^p < 0.01). **(C,D)** Locomotor sensitization. **(C)** Effects of acute and chronic (7 days) treatments with TAT-Sc (300 pmoles, i.c.v.), SKF 83959 (1.5 mg/kg), TAT-D1 peptide (300 pmoles, i.c.v.) and cocaine (10 mg/kg, i.p.) on locomotion. The locomotor sensitization induced by cocaine was abolished by SKF 83959 co-treatment. Animals treated with cocaine plus TAT-D1 exhibited significantly higher locomotor activity compared to cocaine-treated animals. **(D)** Injection of a subthreshold dose of cocaine (5 mg/kg) did not affect the basal locomotor activity of animals previously treated with repeated (7 injections) saline, TAT-Sc, and SKF 83959, but significantly increased the locomotor activity of cocaine-treated animals (10 mg/kg, i.p.), indicating expression of sensitization. In response to the cocaine injection at a subthreshold dose, a sensitized locomotor phenotype was observed in animals previously treated with repeated TAT-D1. The expression of locomotor sensitization was abolished by SKF 83959 and enhanced by TAT-D1. Data in **(C,D)** represent means ± SEM of *n* = 8–10 rats/group. (^*^p < 0.05, ^**^p < 0.01: ^***^p < 0.001: compared to Saline; ^##^*p* < 0.01: compared to Cocaine). **(E)** Cocaine self-administration (SA) under the FR5 schedule. Rats were trained to self administer cocaine intravenously. The animals exhibited steady cocaine self-administration behavior over 2 h sessions following training. SKF 83959 dose-dependently reduced the number of active lever presses and total cocaine infusions. Data in E represent means ± SEM of *n* = 15–16 rats/group. (^**^p < 0.01, ^***^p < 0.001: compared to Vehicle). **(F,G)** Drug-induced reinstatement. A single injection of saline or SKF 83959 did not reinstate the SA behavior. A priming dose of cocaine (10 mg/kg, i.p.) reinstated the SA behavior as indicated by lever presses, which was abolished by co-administration of SKF 83959 at the 0.5 mg/kg **(F)** and 1.5 mg/kg **(G)** doses. **(H)** A single injection of TAT-Sc, TAT-D1 or cocaine at a subthreshold dose (5 mg/kg) did not reinstate the SA behavior, whereas the SA behavior was reinstated by a priming dose of cocaine (10 mg/kg). Pre-treatment with TAT-D1 facilitated the reinstatement of SA behavior induced by a subthreshold dose of cocaine and further enhanced the reinstatement induced by a priming dose of cocaine. Data in **(F–H)** represent means ± SEM. of *n* = 10–12 rats/group. (^**^p < 0.01: compared to Veh and TAT-Sc; ^###^*p* < 0.05: compared to Cocaine). **(I,J)** Cue-induced reinstatement. Presentation of animals to the light cue associated with cocaine was sufficient to reinstate the SA behavior, which was abolished by SKF 83959 at the 1.5 mg/kg **(J)** dose but not at 0.5 mg/kg **(I)**. Data in **(I,J)** represent means ± SEM. of *n* = 10–12 rats/group. (^**^p < 0.01: compared to no cue).

Rats conditioned to cocaine (10 mg/kg, i.p.) exhibited CPP [ANOVA; *F*_(2, 21)_ = 5.00; *p* = 0.017, followed by *t*-test *post-hoc*: Table 1 for detailed statistics], in agreement with previously published data at this cocaine dose (Beninger et al., 1981; Horger et al., 1999), spending approximately 65% of time in the drug-paired chamber [Figure 4A, 1st and 2nd set of bars, Coc: *t*_(8)_ = −2.52; *p* = 0.036]. However, the acquisition of cocaine-induced CPP was blocked when conditioning occurred in the presence of SKF 83959 [Figure 4A, 3rd set of bars, Coc+SKF: *t*_(7)_ = 0.97; *p* = 0.364]. Rats administered the TAT-D1 peptide prior to each injection of cocaine spent almost 75% of their time in the drug-paired chamber on the test day [Figure 4A, 4th set of bars, Coc+TAT-D1: *t*_(6)_ = −3.28; *p* = 0.017], thus showing cocaine-induced CPP, with increased statistical confidence level [ANOVA; *F*_(2, 21)_ = 5.00; *p* = 0.017].

Not only the acquisition but also the expression of cocaine CPP was modulated by the D1-D2 heteromer [Figure 4B, ANOVA; *F*_(3, 28)_ = 3.26; *p* = 0.036], since cocaine-induced CPP was abolished by a single injection of SKF 83959 on the test day in animals that were conditioned with cocaine [Figure 4B, 3rd and 4th set of bars, Coc/Veh: *t*_(8)_ = −3.56; *p* = 0.007; Coc/SKF59: *t*_(8)_ = 0.49; *p* = 0.635]. A single injection of SKF 83959 administered on the test day had no effect on place conditioning in rats given saline during the conditioning phase [Figure 4B, 1st and 2nd sets of bars, Sal/SKF59: *t*_(7)_ = −0.38; *p* = 0.713], which would be expected since the D1-D2 receptor heteromer activation was not associated with a particular environment.

These results suggested that activating the D1-D2 heteromer blocked the cocaine-induced CPP during the phases of acquisition and expression, whereas, blocking the D1-D2 heteromer induced CPP and it may have potentiated cocaine-induced CPP.

#### Modulation of other aspects of cocaine addiction-like behaviors by the D1-D2 heteromer

##### Locomotor sensitization

Acute or repeated (daily for 7 days) administration of SKF 83959 (0.4 mg/kg) or TAT-D1 alone (300 pmoles, i.c.v) to rats had no significant effect on locomotion compared to saline controls. Acute administration of cocaine (10 mg/kg, i.p., Figure 4C, Acute) increased locomotor responding, an effect that was exacerbated by pretreatment with TAT-D1. Repeated cocaine treatment induced a progressive increase in locomotor responding such that animals were significantly more active after Injection 7 compared with Injection 1 (Figure 4C, Chronic), indicative of the successful development of cocaine-induced locomotor sensitization. SKF 83959 pretreatment abolished the development of locomotor sensitization to cocaine without altering the locomotor-stimulating effect of the drug (Statistics below and Table 1). In contrast, repeated pretreatment with TAT-D1 significantly augmented the locomotor response to repeated injections of cocaine (*p* < 0.001), suggesting that the disruption of the D1-D2 heteromer enhanced the cocaine-induced locomotor sensitization [ANOVA Repeated Measures: Treatment Effect: *F*_(5, 44)_ = 36.75, *p* < 0.0001; Treatment × Injection: *F*_(30, 264)_ = 4.35, *p* < 0.0001, ANOVA Day 1: *F*_(5, 44)_ = 9.87, *p* < 0.0001, Day 7: *F*_(5, 44)_ = 31.21, *p* < 0.0001].

##### Response to subthreshold cocaine

To provide further evidence that inhibition of the D1-D2 heteromer could potentiate cocaine-induced behaviors, rats that were pretreated for 7 days with saline, TAT-scrambled peptide (TAT-sc, 300 pmoles, i.c.v.), TAT-D1 (300 pmoles, i.c.v.) or cocaine (10 mg/kg, i.p.) were administered a sub-threshold dose (5 mg/kg, i.p.) of cocaine (Horger et al., 1999) on the test day and locomotor activity was examined [Figure 4D, ANOVA Treatment Effect: *F*_(6, 52)_ = 18.83, *p* < 0.0001, and Table 1]. Repeated treatment with SKF 83959 or the TAT-scrambled peptide had no effect on the locomotor activity induced by cocaine challenge compared to the saline-treated animals. Animals with prior repeated exposure to the TAT-D1 peptide alone exhibited significantly higher locomotor activity in response to cocaine challenge compared to the saline control (*p* < 0.05), indicating that TAT-D1 pretreatment induced a robust sensitized locomotor response to the sub-threshold dose of cocaine (5 mg/kg, ip) on the test day. This effect was not significantly different from that of the cocaine (10 mg/kg, i.p) sensitized rats (Figure 4D), which showed a successful expression of locomotor sensitization to cocaine (*p* < 0.05 and *p* < 0.001, vs. Saline group, respectively). Interestingly, prior SKF 83959 treatment with cocaine abolished, while prior TAT-D1 peptide pre-treatment with cocaine significantly enhanced (*p* < 0.001 vs. Cocaine group), the expression of locomotor sensitization to cocaine, suggesting that activation of the D1-D2 heteromer abolished, while its disruption enhanced the expression of cocaine-induced locomotor sensitization [ANOVA Treatment Effect: *F*_(6, 52)_ = 18.83, *p* < 0.0001].

##### Cocaine self-administration

Animals were trained to self-administer cocaine intravenously, and exhibited a steady increase in the number of active lever presses and the number of cocaine infusions, until the latter plateaued at approximately 25 infusions per 2 h session under the FR5 schedule of reinforcement. After stabilization of SA, three different doses of SKF 83959 were tested (0.05, 0.5, and 1.5 mg/kg) to examine the dose-response relationship between D1-D2 heteromer stimulation and cocaine SA behavior under the FR5 schedule [Figure 4E, ANOVA Within Subjects Effect of Dose: *F*_(3, 25)_ = 3.62, *p* = 0.030]. Pre-treatment with SKF 83959 dose-dependently reduced the number of active lever presses and the number of infusions that was significant at the 0.5 mg/kg (*p* < 0.05) and 1.5 mg/kg (*p* < 0.01) doses. These results suggested that activation of the D1-D2 heteromer dose-dependently blocked cocaine self-administration.

##### Reinstatement of cocaine self-administration

The rats then underwent extinction training over 25 days, during which the number of active lever presses steadily decreased and was eventually stabilized at 15 lever presses per 2 h session. Following extinction training, two separate groups of animals were used to examine the effect of D1-D2 stimulation on cocaine and cue-induced reinstatement using SKF 83959 0.5 mg/kg and 1.5 mg/kg doses. A third group of animals was used to examine the effect of D1-D2 heteromer inactivation on cocaine-induced reinstatement.

A priming injection of cocaine at 10 mg/kg successfully reinstated the cocaine SA behavior as indicated by a significant increase in the number of active lever presses in cocaine-primed animals compared to the vehicle-treated controls [Figures 4F,G, ANOVA Within Subjects Effect of Treatment: *F*_(5, 65)_ = 11.99, *p* < 0.0001]. SKF 83959 co-treatment at 0.5 mg/kg (Figure 4F) and 1.5 mg/kg (Figure 4G) abolished cocaine-induced reinstatement of cocaine SA behavior, while SKF 83959 by itself (without cocaine priming) had no effect. In contrast, co-treatment with TAT-D1 peptide (Figure 4H) with the priming cocaine injection at a subthreshold dose, 5 mg/kg, and the usual priming dose, 10 mg/kg, further significantly enhanced the number of active lever presses induced by the cocaine injections [ANOVA Within Subjects Effect of Treatment: *F*_(5, 65)_ = 11.99, *p* < 0.0001].

These results suggested that activation of the D1-D2 heteromer abolished, whereas blockade of the heteromer facilitated cocaine reinstatement.

Similar to cocaine priming, the presentation of the light cue that was associated with cocaine delivery during cocaine SA training was also able to reinstate cocaine SA behavior [Figures 4I,J, *t*_(10)_ = 3.29, *p* < 0.01 and *t*_(9)_ = 3.70, *p* < 0.01], an effect abolished by SKF 83959 treatment at the 1.5 mg/kg dose (Figure 4J) but not the 0.5 mg/kg dose (Figure 4I). The inactive lever presses were not affected by any of the drug treatments. It should be noted that SKF 83959-treated animals exhibited otherwise normal behaviors such as sniffing and exploring that were comparable to vehicle-treated animals, and thus the reduction in active lever responses following SKF 83959 administration was not due to motor impairment. On the contrary, acute SKF 83959 treatment actually enhanced basal locomotion (Perreault et al., 2010).

These results suggested a role for the D1-D2 heteromer in modulating cocaine- and cue-induced reinstatement, another aspect of addiction to cocaine.

### Signaling pathways involved in D1-D2 heteromer modulation of cocaine-induced behaviors: DARPP-32, pERK, and ΔFosB

#### Activation of the D1-D2 heteromer enhanced Thr75-DARPP-32 phosphorylation in cocaine-treated rats

**Figure 5:**
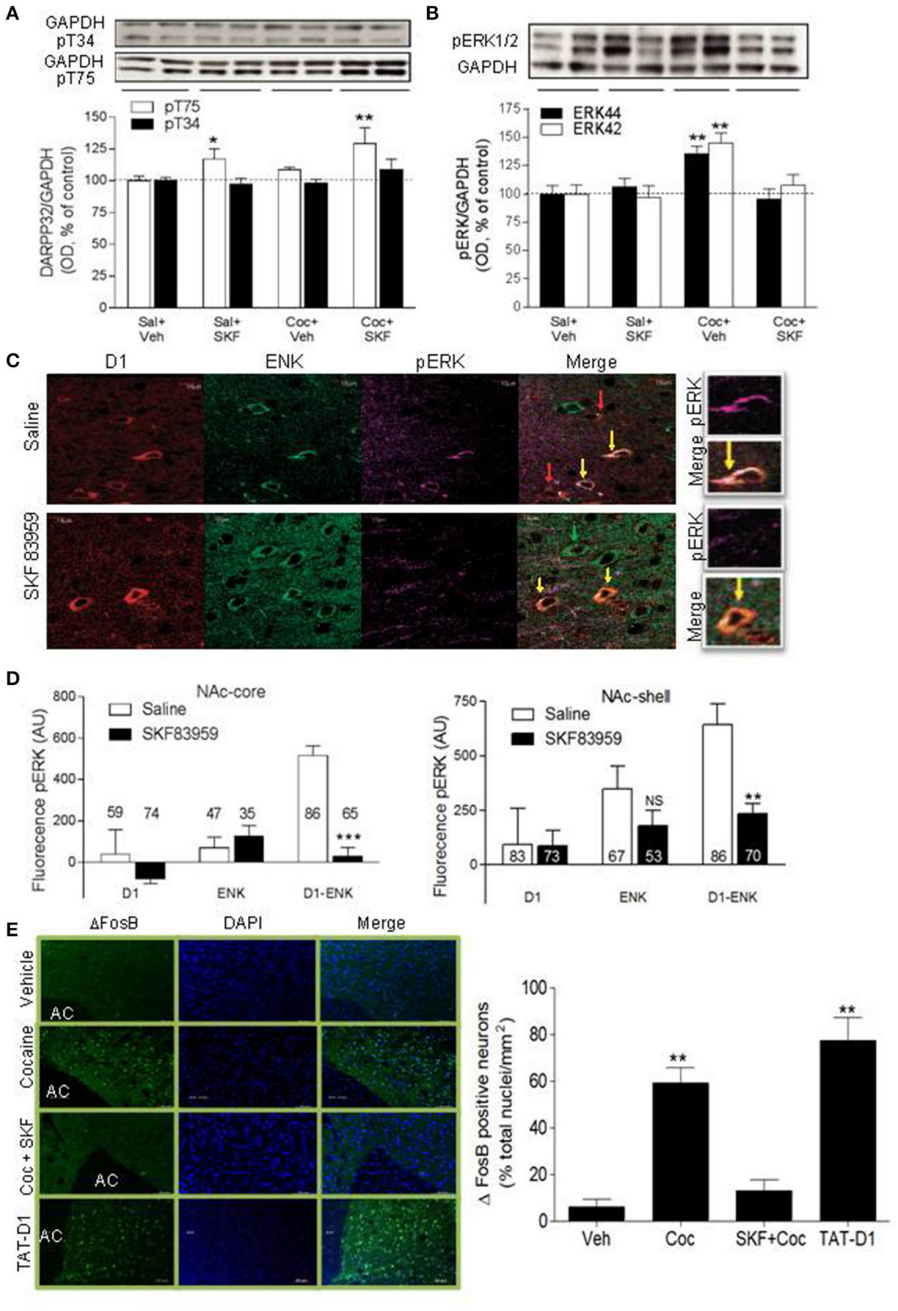
Signaling pathways involved in D1-D2 heteromer modulation of cocaine-induced behaviors: DARPP-32, ERK and ΔFosB. **(A)** Representative immunoblots of pT34-DARPP-32 (top panel) or pT75-DARPP-32 (lower panel) obtained from NAc of rats conditioned with saline or cocaine (10 mg/kg, i.p.) and injected on the test day with saline or SKF 83959. Loading controls (GAPDH) are shown. Quantification of pT34- and pT75-DARPP-32 immunoblots is shown. **(B)** Representative immunoblot of pERK44/42 obtained from the NAc of rats conditioned as in **(A)** is shown. Quantification of pERK44/42 immunoblots obtained from all animals is shown. Results in **(A,B)** represent the mean ± SEM from 8 to 9 rats/condition. ^*^p < 0.05 and ^**^p < 0.01 represent significant differences from control. **(C)** Representative confocal images of pERK assessed in the three types of MSNs: D1R-only (red arrow), Enk-only (D2R, green arrow) or D1R and ENK (D1-D2 heteromer)-expressing neurons (yellow arrow) in MSNs from NAc of saline- or SKF 83959-treated rats. **(D)** Quantification of pERK fluorescence in MSNs in the NAc. Results are the mean ± SEM of data after removing the non-specific background (*n* = number of MSNs from *N* = at least 3 rats/condition). (^**^p < 0.001; ^***^p < 0.0001). **(E)** Representative immunohistochemistry images and their quantification obtained using an antibody against ΔFosB and a secondary antibody conjugated to Alexa-488. Nuclei are stained by DAPI. Rats were treated for 7 days with cocaine (10 mg/kg, i.p.) without or with co-injection of SKF 83959 (1 mg/kg, s.c.). Disrupting the heteromer by repeated injections of TAT-D1 had the same effect as repeated injections of cocaine. Results are means ± SD obtained by the analysis of *n* = 1,500–1,700 neurons from the NAc of *N* = 3 rats/condition.

We tested if the blockade of cocaine-induced CPP by D1-D2 heteromer involved the modulation of DARPP-32. The effect of SKF 83959 on cocaine-induced DARPP-32 signaling was tested in animals conditioned to saline or cocaine 10 mg/kg (three injections in 6 days, D-S-D-S-D-S). On the test day, the animals were injected with saline or SKF 83959. In comparison to saline-treated animals (Figure 5A, 1st set of bars), SKF 83959-treated rats (Figure 5A, 2nd set of bars) showed an increase of pT75-DARPP32 by western blot but not of pT34 in NAc [ANOVA followed by Benferroni's multiple comparisons, *F*_(3, 33)_ = 6.93, *p* = 0.001; and detailed statistics in Table 1], while cocaine treatment (Figure 5A, 3d set of bars) resulted in a small but non-significant increase in pT75. Animals conditioned with cocaine and injected with SKF 83959 (Figure 5A, 4th set of bars) showed a significant increase in pT75 in NAc but no effect on pT34. These results suggested that D1-D2 heteromer activation might have blocked the expression of cocaine-induced CPP through a mechanism involving increased pT75-DARPP-32.

#### Activation of the D1-D2 heteromer attenuates cocaine-induced ERK activation

Further, cocaine (Figure 5B, 3d set of bars) induced 46 ± 7% increase in NAc pERK compared to saline-treated animals [Figure 5B, 1st set of bars, ANOVA followed by Benferroni's test for multiple comparisons, ANOVA pERK42 *F*_(3, 33)_ = 9.541, *p* = 0.0001; ANOVA pERK44: *F*_(3, 33)_ = 9.314, *p* = 0.0002; detailed statistics for pERK42 and pERK44 in Table 1], an effect abolished in animals injected with SKF 83959 (Figure 5B, 4th set of bars). These results suggested that the inhibition of cocaine-induced CPP by D1-D2 heteromer stimulation involved inhibition of the pERK pathway. The effects of stimulating the D1-D2 heteromer on pERK were then tested in rats injected with saline or SKF 83959 (1.5 mg/kg, s.c.). Western blot analysis in NAc showed no effect of treatment with SKF 83959 in a time course up to 90 min. However, immunohistochemistry (Figure 5C) and its quantification (Figure 5D) revealed after 15 min of treatment, a clear difference in pERK depending on both the cell type [D1R, ENK (D2R) or D1R-Enk] and the region examined. In NAc-core [D1-Enk: *t*_(150)_ = 6.430; *p* < 0.0001 D1-only: *t*_(131)_ = 0.12; *p* = 0.86 Enk-only: *t*_(80)_ = 0.165; *p* = 0.90] and NAc-shell [D1-Enk: *t*_(148)_ = 6.691; *p* < 0.0001 D1-only: *t*_(127)_ = 0.0609; *p* = 0.9515 Enk-only: *t*_(77)_ = 1; *p* = 0.2399], following SKF 83959 injection pERK decreased in neurons coexpressing D1R and ENK (i.e., expressing D1-D2 heteromer, yellow arrow) both in the core and the shell subregions, while MSNs expressing D1R (red arrow) or D2R alone (green arrow) showed no significant change. It was noted that basal pERK was significantly higher in D1-ENK MSNs in NAc core and shell compared to ENK only- or D1R only-expressing neurons (Figures 5C,D). Such a difference in basal pERK was not observed in the CPu, where the heteromers are scant or absent (Hasbi et al., 2009; Perreault et al., 2010) and no significant effect was observed in any of the three types of neurons in CPu after treatment with SKF 83959.

The results *in vivo* suggested that activation of the D1-D2 receptor heteromer-mediated signaling pathway led to inhibition of pERK activity selectively in the D1-D2 heteromer-expressing neurons in the NAc shell and core but this effect was not detectable by WB of NAc tissue.

These data combined suggested that the D1-D2 heteromer-induced inhibition of cocaine-elicited effects involved an augmentation of Thr75-DARPP-32 phosphorylation and an inhibition of cocaine-induced ERK activation in the NAc.

#### Activation of the D1-D2 heteromer attenuates cocaine-induced ΔFosB expression

Repeated injections of cocaine increased ΔFosB in the NAc (Figure 5E, Supplementary Figure 7B), in accordance with previous reports (Nestler, 2005, 2008). This effect was blocked by stimulation of the D1-D2 heteromer in animals co-treated with cocaine and SKF 83959. Stimulation of the D1-D2 heteromer without cocaine injection had no significant effect on ΔFosB. These results showed that stimulation of D1-D2 heteromer opposed the cocaine-induced increase in ΔFosB and suggested that D1-D2 heteromer activation may prevent some neuroadaptations associated with cocaine use. Similar to cocaine treatment, disruption of the D1-D2 heteromer by repeated administration of the TAT-D1 peptide, and not the control TAT-scrambled peptide (not shown), produced an increase in ΔFosB (Figure 5E), suggesting that the D1-D2 heteromer had a tonic inhibitory effect on mechanisms controlling ΔFosB expression, which was alleviated by the disruption of the receptor complex.

### Electrophysiological study: activation of the D1-D2 heteromer attenuates cocaine-induced increase in spontaneous local field potentials

**Figure 6:**
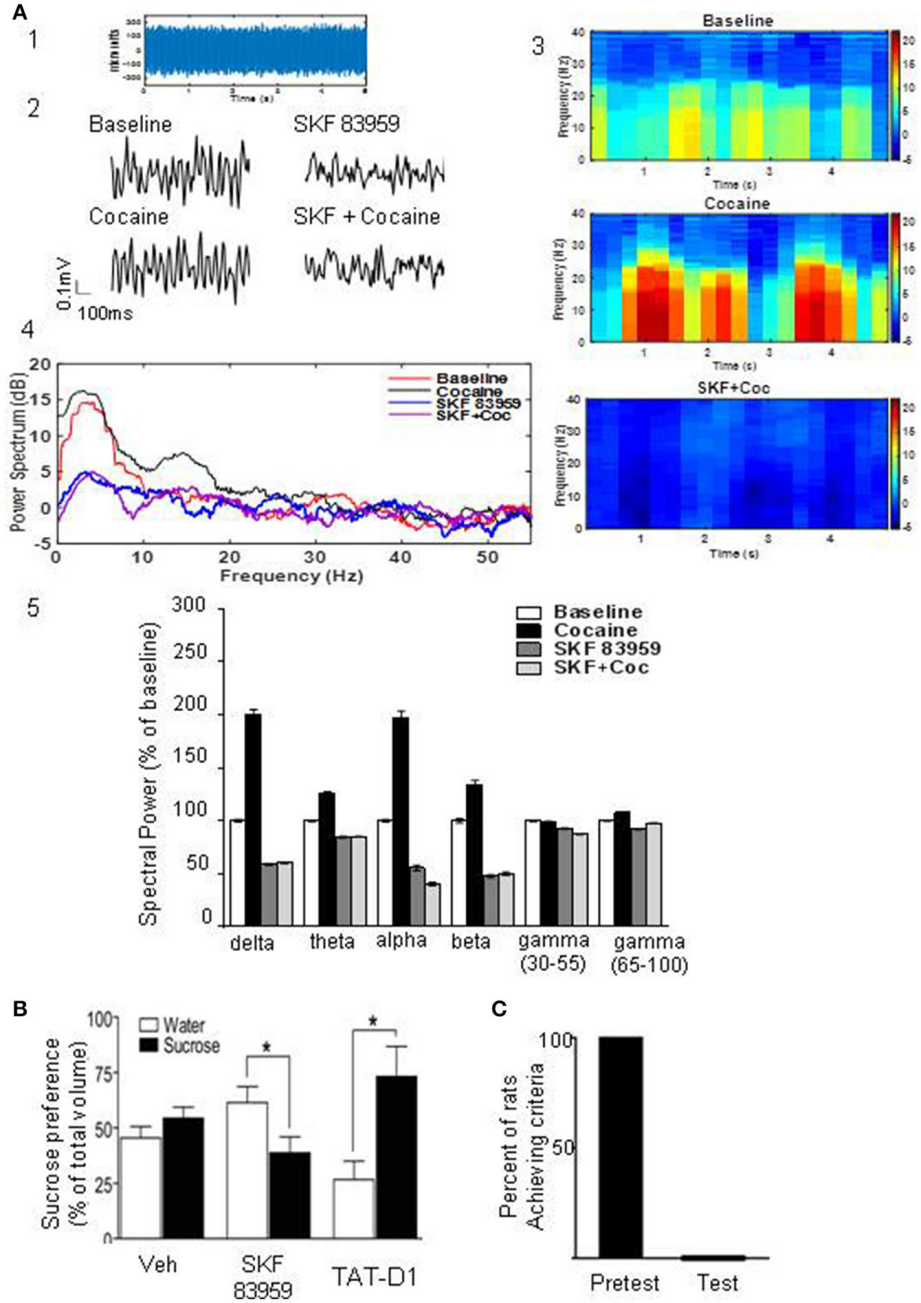
Heteromer D1-D2 activation inhibits cocaine-induced changes in LFPs and modulates food reward. **(A)** Analysis of local field potentials (LFPs) from a sample 5 s epoch of baseline LFP recordings derived from rat NAc **(A1)**. Representative 100 ms LFP amplitude recordings of baseline and recordings derived from rat NAc treated with cocaine, SKF 83959 or both drugs **(A2)**. Spectrograms depicting time-frequency analysis showing the relative change in power of specific frequencies across the 5 s epoch of rats treated with cocaine or cocaine + SKF 83959 in comparison to the baseline **(A3)**. Representative power spectrum showing acute cocaine induced increase in spectral power at lower frequencies (<30 Hz) which was suppressed by pretreatment with SKF 83959 **(A4)**. Drug-induced changes in mean spectral power at select frequencies are also shown **(A5)**. **(B)** Sucrose preference test. A single injection of SKF 83959 in a two bottle free choice paradigm significantly decreased the proportion of sucrose consumed but had no effect on water consumed (1st and 2nd set of bars). Administration of TAT-D1 (300 pmoles i.c.v) significantly increased the proportion of sucrose consumed (3rd set of bars). Results represent the mean ± SEM from 8 to 9 rats/condition. ^*^p < 0.05 represents significant difference from control. **(C)** Activation of the D1-D2 heteromer abolished the motivation of rats to search for and consume a palatable sweet treat. Rats that had been successfully trained (pretest) were administered a single injection of SKF 83959 (1.5 mg/kg s.c.), placed in the radial arm maze and the number of trials required to reach the set criteria (11 consecutive correct choices) were documented. In each animal tested (Test), SKF 83959 abolished the motivation of the animals to search for and consume the food reward.

We investigated the effects of activating the D1-D2 heteromer on neuronal activity in the NAc (Figure 6A). It has been previously demonstrated that cocaine administration influences neuronal oscillatory patterns in NAc (McCracken and Grace, 2013). We therefore evaluated the effects of D1-D2 heteromer activation on cocaine-induced changes in spontaneous local field potential (LFP) activity in this region. Baseline LFP oscillatory recordings were taken from NAc of anesthetized rats. LFPs from a sample 5s epoch at baseline were recorded from rat NAc (Figure 6A1). Activation of D1-D2 heteromer by SKF 83959 (1.5 mg/kg s.c.) decreased baseline LFP amplitude as well as after acute cocaine (10 mg/kg i.p.) treatment, as depicted by the 100 ms recordings (Figure 6A2). Acute administration of cocaine increased spontaneous LFP power compared to baseline as shown by the spectrograms depicting time-frequency analysis (Figure 6A3), the representative power spectrum (Figure 6A4), and the histogram summarizing the changes in mean spectral power at select frequencies (Figure 6A5). In contrast, SKF 83959 decreased spontaneous LFP power. These effects were particularly evident at lower frequencies (1–30 Hz). Consistent with the behavioral data, pretreatment with SKF 83959 reversed cocaine-induced changes in spontaneous LFP power at all frequencies as shown in the representative power spectrum (Figure 6A3), the spectrogram time-frequency analysis (Figure 6A4), and summarized in the histogram (Figure 6A5).

### Effects of the D1-D2 heteromer on natural rewards

We next investigated if these inhibitory effects of the D1-D2 heteromer on cocaine-induced reward mechanisms could be generalized to natural rewards using sucrose consumption and the motivation to work for a palatable food reward.

A single injection of SKF 83959 immediately before exposure to a two bottle free choice paradigm of water and sucrose (1%) significantly decreased the proportion of sucrose consumed but had no effect on water consumed (Figure 6B). In contrast, TAT-D1 (300 pmoles, i.c.v) significantly enhanced the proportion of sucrose consumed, suggesting that disruption of the D1-D2 heteromer enhanced the rewarding aspects of sucrose (Figure 6B).

In order to assess the role of the D1-D2 heteromer in the motivation to work for a food reward, the rewarding properties of a palatable sweet treat were utilized (Figure 6C). Food-restricted rats (12 h) were habituated to the radial arm maze and trained to consume treats placed within the maze in a specific time frame of 15 min. Once the rats (*n* = 16) met these established criteria (in 6–10 days), they underwent a response test, in which each animal had to learn to turn in a specific direction in the maze to receive the food reward. Rats that had been successfully trained (Figure 6C, pretest) were administered a single injection of SKF 83959 (1.5 mg/kg s.c.), placed in the maze and the number of trials required to reach the set criteria (11 consecutive correct choices) were documented. In each animal tested (Figure 6C, test), SKF 83959 abolished the motivation to search for and consume the food reward, in spite of individual trials extending well over 60–90 min. As documented previously (Perreault et al., 2012), SKF 83959 did not induce catatonia or induce locomotor inactivation. Therefore, the present findings showed that activation of the D1-D2 heteromer effectively abolished both the consumatory and motivational aspects of a palatable reward.

These data demonstrated the tonic inhibitory effect exerted on consumatory reward by the D1-D2 receptor heteromer, activation of which abolished the motivation to seek and consume palatable food while its disruption led to disinhibition of the negative control over reward pathways with augmentation of reward-seeking behavior.

## Discussion

The present findings show conclusively that the dopamine D1-D2 receptor heteromer exists in the NAc of rat and non-human primates and provide strong evidence for a physiologic regulatory mechanism in which there is a fundamental role for the receptor heteromer in rat NAc in applying tonic and active blockade of drug and natural reward mechanisms. Essentially, we showed that the D1-D2 heteromer modulated all aspects of cocaine-induced behaviors and key biochemical parameters. Based on the inhibition of SKF 83959 effects by the selective peptide TAT-D1, our data showed that activation of the D1-D2 heteromer induced CPA, abolished cocaine-induced CPP, cocaine-induced locomotor sensitization, cocaine self-administration and cocaine-induced, as well as cue-induced, reinstatement of drug seeking and palatable food reward. In contrast, disruption of the D1-D2 heteromer was in itself rewarding in the CPP paradigm, enhanced cocaine-induced on CPP, locomotor sensitization and reinstatement and increased sucrose consumption. We also show that at least three important biochemical mechanisms involved in cocaine actions were abolished by activation of the D1-D2 heteromer, through Cdk5-mediated increase in Thr75-DARPP-32 phosphorylation with a concomitant decrease in Thr34-DARPP-32 phosphorylation, inhibition of cocaine-induced ERK1/2 phosphorylation, and inhibition of cocaine-induced ΔFosB expression.

Firstly, the existence of a dopamine D1–D2 heteromer has been challenged (Frederick et al., 2015), in a study where PLA failed to be detected between D1R and D2R despite the numerous lines of evidence demonstrating colocalization and/or physical interaction between D1R and D2R in the mammalian striatum, notably in the ventral striatum (Meador-Woodruff et al., 1991; Weiner et al., 1991; Lester et al., 1993; Lee et al., 2006; Bertran-Gonzalez et al., 2008; Hasbi et al., 2009; Matamales et al., 2009; Pei et al., 2010; Perreault et al., 2010; Gagnon et al., 2017; Rico et al., 2017). In direct contrast to that report (Frederick et al., 2015), and using the same two sets of antibodies, we demonstrated D1-D2 heteromer complexes by PLA in adult mouse, rat and monkey striatum. The PLA signal was clearly observed at the cell surface, with no nuclear labeling in line with the non-existence of D1R or D2R in cell nuclei. This is in contrast to the PLA results from Frederick et al. (2015), who showed high levels of non-specific nuclear staining, suggesting that there were significant methodological differences from our study. Our PLA results are in agreement with recently published PLA data in macaque, which showed clearly the presence of the D1-D2 heteromer in the non-human striatum, with a higher incidence in the ventral than in the dorsal striatum (Rico et al., 2017). Furthermore, in contrast to Frederick et al. (2015), who reported that only around 2% MSNs co-express D1R and D2R in the ventral striatum of the double BAC Drd1a-TdTomato/Drd2-GFP transgenic mice, a recent and more detailed study in this double transgenic mouse model showed that colocalization between D1R and D2R was 14% in the NAc-shell and 7% of the NAc-core (Gagnon et al., 2017). In mouse NAc, both in wild type and the D5^−/−^ KO mice used as controls, the number of neurons with PLA signal was estimated to be 14–16%, which is consistent with the rate of colocalization between D1R and D2R in the ventral striatum, notably the shell subregion (Gagnon et al., 2017). Also, the data from the double transgenic mice as well as the PLA in wild type and D5^−/−^ KO mice are in line with estimations from D1-GFP or D2-GFP BAC transgenic mice (Bertran-Gonzalez et al., 2008; Matamales et al., 2009; Gangarossa et al., 2013). In rat, our present *in situ* PLA and *in situ* FRET data are in line with previous estimations of MSNs expressing the heteromer (20–25% in the NAc) obtained by FRET on rat brain slices (Hasbi et al., 2009; Perreault et al., 2010). These numbers from rat NAc are higher than those estimated in mice NAc but lower than the estimations of heteromers in the striatum of macaque obtained by PLA (Rico et al., 2017), suggesting a species difference.

Activation of the D1-D2 receptor heteromer resulted behaviorally in place aversion and negatively impacted the reward related to cocaine or natural reinforcers and induced a diminished interest and motivation to seek palatable food. Disruption of the heteromer by TAT-D1 blocked these SKF-83959-induced effects indicating involvement of heteromer activity in the SKF 83959 effects. Remarkably, administration of TAT-D1 alone induced CPP and enhanced sucrose preference indicating that basal D1-D2 receptor heteromer activity had tonic inhibitory effects on limiting reward perception, which were relieved by D1-D2 disruption by TAT-D1. This role was clearly exhibited when disruption of the D1-D2 heteromer facilitated the development of reinstatement of cocaine SA at a subthreshold dose of cocaine and in another related study (Perreault et al., 2016), where similar conclusions were drawn. Thus, the present data revealed a previously unknown role of the dopamine system, through the D1-D2 receptor heteromer, to exert tonic and active inhibitory effects on limiting reward mechanisms, analogous to inducing anhedonia with a markedly diminished interest and motivation to seek consumatory and drug-induced reward. Therefore, it appears that it is not simply a lack of reward stimulation that results in anhedonia but the activation of a specific inhibitory/aversive pathway, in which the D1-D2 heteromer seems to play a major role. Consequently, the reward experienced would be the result of the balance between stimulation of both rewarding and aversive dopaminergic mechanisms.

The present data emphasize a central and direct role for Cdk5 and DARPP-32 in the D1-D2 heteromer-mediated signaling pathway responsible for the inhibitory effects on reward. A single injection of SKF 83959 induced an increase in Thr75-DARPP-32 phosphorylation and a decrease in Thr34-DARPP-32 phosphorylation, exclusively in neurons coexpressing D1R and D2R as observed by IHC and WB. Repeated D1-D2 heteromer activation (3 alternate day injections) resulted in place aversion, which was mediated through the action of Cdk5 to phosphorylate Thr75-DARPP-32 as indicated by the use of the Cdk5 inhibitor roscovitine.

Further, activation of the D1-D2 heteromer inhibited both the development and expression of cocaine-induced CPP through a mechanism involving DARPP-32. Repeated co-injection of cocaine and SKF 83959 during the acquisition phase blocked cocaine-induced CPP development, whereas, a single injection of SKF 83959 on the final test day blocked cocaine-induced CPP expression. The single injection of SKF 83959 on the test day was able to enhance Thr75-DARPP-32 phosphorylation in animals pretreated with cocaine. Following repeated cocaine injections, a tendency to higher phosphorylation of Thr75-DARPP-32 was observed in animals exhibiting cocaine-induced CPP (3 alternate day injections). These results suggested that the heteromer was able to counter cocaine-induced effects through activation of a Cdk5-Thr75-DARPP-32 pathway in the NAc. Thus, these findings established that the dopaminergic system was capable of mediating a direct inhibitory/aversive effect through the activation of the D1-D2 receptor heteromer-induced and Cdk5-mediated Thr75-DARPP-32 activation, which also resulted in decreased phosphorylation of Thr34, probably through an inhibition of the cAMP/PKA signaling cascade, since it was previously shown that activation of Thr75-DARPP-32 converts DARPP-32 into an inhibitor of the PKA pathway (Bibb et al., 1999, 2001; Nishi et al., 2000; Takahashi et al., 2005; Benavides et al., 2007). This D1-D2 receptor heteromer-mediated mechanism, able to abolish cocaine-induced effects, may function as a regulatory mechanism able to block cocaine reward and therefore cocaine-seeking. Activation of the D1R/cAMP/PKA/Thr34-DARPP-32 pathway was shown to lead to the inhibition of PP-1, and activation of the ERK cascade (Greengard et al., 1999; Valjent et al., 2000, 2006; Svenningsson et al., 2004; Bertran-Gonzalez et al., 2008). While cocaine is known to elevate pERK to mediate its actions, which we confirmed, SKF 83959 abolished this effect, suggesting that the mechanism by which D1-D2 heteromer activation inhibited cocaine-CPP also involved an inhibition of the cAMP/PKA-ERK pathway.

An important and critical step for the progression in the cocaine addiction cycle is mediated by ΔFosB, with repeated cocaine exposure resulting in its sustained expression (reviewed in Nestler, 2008) and leading to multiple molecular and structural alterations. Our present results confirmed the cocaine-induced increase in ΔFosB expression, and also showed that activation of the D1-D2 heteromer was able to abolish the cocaine-induced increase in ΔFosB. Importantly, the D1-D2 heteromer seemed to exert a tonic inhibitory effect on the generation of ΔFosB, since simple disruption of the D1-D2 heteromer chronically led to an elevation in ΔFosB expression, confirming previous observations (Perreault et al., 2016) and providing another piece of evidence for the tonic inhibitory role played by the D1-D2 heteromer.

It has been previously demonstrated that cocaine administration influences neuronal oscillatory patterns in NAc (McCracken and Grace, 2013). Our electrophysiological data showed that acute administration of cocaine increased the power of spontaneous LFPs from NAc compared to baseline. However, activation of D1-D2 heteromer was able to block these cocaine-induced changes in spontaneous LFP power at all frequencies, which is consistent with the behavioral data showing the inhibitory effects of the D1-D2 heteromer on cocaine-induced effects.

These behavioral, biochemical and electrophysiological effects point to the conclusion that the D1-D2 receptor heteromer exerts a key role in the modulation of basal as well as activated reward mechanisms, to block the initiation, progression and relapse to cocaine seeking and sensitization and perhaps to other psychostimulants, as indicated by effects on amphetamine-induced locomotor sensitization (Shen et al., 2015). We established that D1-D2 heteromer activation modulated DARPP-32 activity and blocked cocaine induced ERK phosphorylation and ΔFosB expression. We demonstrated the effects of heteromer activation on phosphorylation of Thr75-DARPP-32 to occur directly in D1-D2 neurons. However the effects of investigator- and self-administered cocaine to enhance ΔFosB expression have been shown to occur in D1 neurons (Moratalla et al., 1996; Kelz et al., 1999; Colby et al., 2003; Zachariou et al., 2006; Winstanley et al., 2007; Perrotti et al., 2008; Lobo et al., 2013). The mechanism(s) by which the changes in D1-D2 heteromer expressing neurons may influence D1R neurons to reverse the cocaine induced changes is not yet established. It is plausible that MSN-MSN interactions (O'Donnell and Grace, 1993; Geldwert et al., 2006; Calabresi et al., 2014) may contribute to that mechanism and/or other players such as BDNF may be involved. We showed that activation of the D1-D2 heteromer induced BDNF expression (Hasbi et al., 2009; Ng et al., 2010; Perreault et al., 2012), which may be released from these neurons and through its receptor, TrkB, shown to be present on both D1R and D2R MSNs (Lobo et al., 2010; Lobo and Nestler, 2011), may influence the signaling in D1R and D2R expressing neurons. Indeed, cocaine-induced locomotor activity and the induction of cocaine CPP were enhanced after TrkB deletion from D1R MSNs, but attenuated after deletion from D2R MSNs (Lobo et al., 2010). One can consider that the neurons expressing the D1-D2 heteromer may modulate the activity of the D1R and D2R MSNs via BDNF/TrkB.

Despite a significant amount of evidence linking dopamine function in NAc to aspects of aversive motivational processes, the major focus has been on DA function in rewarding processes (reviewed in Salamone and Correa, 2012). It has been shown that aversive stimuli inhibit dopamine neurons in the VTA (Ungless et al., 2004; Valenti et al., 2011) and optogenetic experiments have shown that the inhibition of dopamine neurons resulting in CPA was mediated by GABA neurons of the VTA through neurotransmission at GABA_A_ receptors (Tan et al., 2012). Further, the stimulation of GABA neurons in the VTA was shown to directly inhibit the activity and excitability of VTA dopamine neurons and also to suppress the release of DA in the NAc (van Zessen et al., 2012). Notably, GABA neurons of the VTA were shown to receive inhibitory afferents from MSNs of the NAc (Xia et al., 2011), which fits well with our data showing that the activation of the DA D1-D2 receptor heteromer in the NAc, notably in the shell subregion, induced enhanced GABA-related protein expression in NAc and VTA, indicative of increased GABA transmission to these regions (Perreault et al., 2012). The neuronal projections of the D1-D2 heteromer-expressing neurons from NAc to VTA and other regions (whether direct or indirect) have not yet been fully characterized. Recent data showed that the activation of inputs to the VTA from the laterodorsal tegmentum or the lateral habenula led to reward or aversion, respectively (Lammel et al., 2011). Taken together, this evidence suggests that the activation of the D1-D2 receptor heteromer may exert its inhibition of reward-related behavior, at least in part, by increasing GABAergic tone at the NAc level, and potentially through its efferents, at the VTA.

The present study describes biochemical and behavioral effects modulated by the dopamine D1-D2 receptor heteromer to exemplify an important counter-regulatory mechanism, which may represent a first line of defense opposing the progressive development of addiction to psychostimulants and drugs of abuse, and also suppressing appetitive reinforcement signals. The tonic as well as active modulation of reward mediated through the D1-D2 receptor heteromer may represent a physiologic basis for anhedonia, by which the dopamine system could exert a brake on the processes that regulate reward perception and appetitive motivation. The D1-D2 receptor heteromer may thus represent an important novel pharmacological target for pathophysiologies involved in drug dependence and addiction, motivational disorders, and dysregulated consumatory behaviors such as hedonic overeating.

## Materials and methods

### Animals

Adult male Sprague-Dawley rats (300–325 g; Charles River, Canada) were used. The rats were housed in pairs and maintained in a 12:12 h light:dark cycle with food and water available *ad libitum*. They were acclimatized for at least 1 week before they were included in studies. Procedures in this study were carried out in compliance with the guidelines described in the Guide to the Care and Use of Experimental Animals (Canadian Council on Animal Care, 1993). The protocol was approved by the University of Toronto Animal Use Protocol Committee.

Frozen striatal tissue from monkey was a generous gift from Dr. R. Tyndale (University of Toronto).

### Drugs

SKF 83959 hydrobromide (Tocris Bioscience) was dissolved in physiological saline containing 5% DMSO and administered subcutaneously. Cocaine hydrochloride (Medisca Pharmaceutique, St Laurent, Canada) was dissolved in saline and given intraperitoneally. For non-drug injections, an equivalent volume of saline/vehicle was used. All drug injections were administered in a volume of 1.0 ml/kg. In rats that received the Cdk5 inhibitor, roscovitine (Sigma Aldrich; 0.2 or 200 nmoles/4 μl, i.c.v. or 25–30 nmoles/0.5 μL intra-accumbal injection), the TAT-scrambled peptide or the TAT-D1 peptide (Genscript; Hasbi et al., 2014; 300 pmoles/4 μl, i.c.v.), the drug or vehicle was administered 15 min prior to SKF 83959 or cocaine injection. Roscovitine was dissolved in physiological saline containing 5% DMSO, whereas the TAT-scrambled peptide and the TAT-D1 peptide were dissolved in sterile water and diluted in physiological saline.

### Antibodies

The primary antibodies used in the present study were raised against: Dopamine D1R (Sigma, D2944, rat); dopamine D2R for IHC and PLA (1st set) (Millipore; AB5084P; rabbit), dopamine D2R for co-IP (Alomone; Rabbit), dopamine D2R for second set PLA (Millipore, ABN 462), phospho-Thr75-DARPP-32 (Cell signaling, 2301s; rabbit), phospho-Thr34-DARPP-32 (Cell Signaling, 2304; rabbit), enkephalin (Millipore, MAB350, mouse), phospho-ERK1/2 (Sigma, E7028; rabbit), ΔFosB (Cell signaling, 14695S, rabbit), GAPDH (Millipore, rabbit).

### Proximity ligation assay (PLA)

To create our PLA probes a rat anti-D1R antibody (Sigma, D2944) was conjugated with a PLUS oligonucleotide (Duolink® *In Situ* Probemaker PLUS DUO92009, Sigma-Olink) and a rabbit anti-D2R (Millipore, AB5084P) antibody with a MINUS oligonucleotide (Duolink® *In Situ* Probemaker MINUS DUO92010, Sigma-Olink) following manufacturer's instructions. The PLA protocol was performed as described by the manufacturer (Duolink®, Sigma-Olink). Briefly, coronal slices from rat brain (25 μm, Bregma:1.6 ± 0.6) or monkey (30 μm) were incubated for 1 h at 37°C with the blocking solution in a pre-heated humidity chamber, followed by incubation with the generated PLA probes described above (final concentration of 60 μg/ml) and washed with buffer A (DUO82047, Sigma-Olink). The PLA signal was detected using the Duolink II *in situ* PLA detection kit (DUO92008, Sigma-Olink) after the ligation-amplification steps. Nuclei were labeled by a DAPI solution included in the last washing step in buffer B × 0.01 (DUO82048, Sigma-Olink). Positive PLA signals were easily identified as red dots around cell bodies (visualized by nuclei staining with DAPI) using confocal Fluoview Olympus microscope (FV 1000) with 40 × or 60 × /1.2 NA objectives. Z-stacks were taken to confirm that PLA signals were localized on cell bodies. Cell counting and analysis of the PLA signal were performed using Imagetool software (Duolink^®;^). The reported percentages are calculated from images taken by the 60 × /1.2 NA objective or magnified portions of these images. Image sizes were [211.735 μm ^*^ 211.735 μm] for the whole images taken by a 60X objective (NA 1.2), [105.468 μm ^*^ 105.468 μm]” for a 2 × zoom and [70.312 μm ^*^ 70.312 μm] for a 3 × zoom of these images. The number of total neurons examined is indicated within the text describing the results (Figure 1A). Appropriate negative control assays were performed to ensure the specificity of the PLA labeling and amplification. These control assays were carried out in the absence of one of the two PLA probes or both, or in the absence of the ligase and/or polymerase. No PLA signal was observed in these conditions (shown as Supplementary Figure 1A).

### Immunohistochemistry

Immunohistochemistry was performed on paraformaldehyde-fixed floating coronal sections (20 μm, Bregma: 1.6 ± 0.6) from perfused rat brain. Sections were blocked (10% goat serum, 1% BSA, 0.1% Triton-X in 0.05M PBS) and then incubated for 60 h at 4°C with primary antibodies in buffer (2% goat serum, 0.01% Triton-X in 0.05M PBS) as previously described (Lee et al., 2004; Hasbi et al., 2009). The primary antibodies used were: anti-phospho-Thr75-DARPP-32 (Cell signaling; 1:400), anti-phospho-Thr34-DARPP-32 (Cell Signaling; 1:400), and anti-total DARPP-32 (Cell Signaling; 1:400), anti-pERK1/2 (Sigma, 1:400), anti-D1R (Sigma, 1:300), anti-ENK (Millipore, mouse, 1:200), and anti-ΔfosB (Cell signaling, 14695S). After three washes with 0.05M PBS the samples were incubated with the appropriate secondary antibody in buffer for 2h at room temperature. The secondary antibodies conjugated to fluorophores (Alexa Fluor 488; Alexa Fluor 350, Alexa Fluor 568) were all purchased from Molecular Probes and used at 1:500. After three washes, the slides were mounted by using a mounting solution (Dako), and the images were acquired by using a confocal Fluoview Olympus microscope (FV 1000) with 40 × or 60 × /1.2 NA objective. Lower magnification images (40×) were used for cell counting. All images were acquired in sequential mode to minimize any bleed-through.

### D1R and D2R colocalization by immunohistochemistry

For experiments of D1R and D2R colocalization, we eliminated any use of secondary antibodies by directly conjugating the anti-D1R antibody (Sigma, D2944) to Alexa Fluor-488, and the anti-D2R antibody (Millipore, AB5084P) to Alexa Fluor-568, accordingly to the manufacturer's instructions (Invitrogen). Coronal sections (16 μm, Bregma:1.6 ± 0.6) from perfused rat brain were blocked (1% BSA, 0.1% Triton-X in 0.05M PBS) and then incubated for 96 h at 4°C with anti-D1-488 (1:200) and anti-D2-568 (1:200). After three washes, the last one containing DAPI, the slides were mounted (Dako), and the images were acquired in sequential mode in the same conditions described above.

### Confocal microscopy fluorescence resonance energy transfer (FRET)

Confocal microscopy FRET analysis and data processing was performed as described previously (Hasbi et al., 2009; Perreault et al., 2010) with the following changes. The directly conjugated anti-D1R-488 and anti-D2R-568 antibodies were used as the FRET donor and acceptor dipoles, respectively. The analysis was performed as previously described using Olympus software, which allows the calculation of energy transfer efficiency (*E*) and the distance (*r*) between the donor and the acceptor molecules. Briefly, eleven control images were acquired for each FRET analysis. The processed FRET (pFRET) images were then generated based on an algorithm in which: pFRET = UFRET − ASBT − DSBT, where UFRET is uncorrected FRET and ASBT and DSBT are the acceptor and the donor spectral bleed-through signals, respectively. The rate of energy transfer efficiency (*E*) and the distance (*r*) between the donor (*D*) and the acceptor (*A*) molecules were estimated by selecting small regions of interest (ROI) using the same images and software, based on the following equations:

(1) $$E\;=\;1-I_{\text{DA}}/[I_{\text{DA}}+\text{pFRET}\times ((\psi_{\text{dd}}/\psi_{\text{aa}})\times (Q_{\text{d}}/Q_{\text{a}}))],$$

where *I*_DA_ is the donor image in the presence of acceptor, ψ_dd_ and ψ_aa_ are collection efficiencies in the donor and acceptor channels, respectively, and *Q*_d_ and *Q*_a_ are the quantum yields. *E* is proportional to the sixth power of the distance (*r*) separating the FRET pair:

*r* = *R*_0_ [(1/*E*) − 1]^1/6^. *R*_o_ is the Förster's distance.

### Co-immunoprecipitation of the D1-D2 heteromer

Protein homogenates (300 μg /each condition) from rat NAc were incubated with an anti-D2R antibody (Rabbit, Alomone) at 4°C overnight under gentle rotation. After adding 40–50 μl of protein G/A, the mixture was further incubated for 1 h. After 3 washes with PBS-Tween, SDS buffer (70 μl) was added, and the immunoprecipitates were incubated for 5 min at 95°C. Proteins were resolved by electrophoresis on 10% polyacrylamide gels under denaturing conditions (SDS-PAGE) and transferred onto nitrocellulose or PVDF membranes (Bio-Rad Laboratories, Hercules, CA, USA) using a semidry transfer system (Invitrogen, Carlsbad, CA, USA). Membranes were incubated in PBS-Tween (PBS-T)/10% nonfat milk for 1 h. After 3 washes in PBS, membranes were incubated with PBS-T/5% nonfat milk containing the anti-D1R antibody raised in rats (Sigma, St. Louis, MO, USA). Membranes were washed once in PBS-T and 2 times in PBS (10 min each) and incubated with the appropriate horseradish peroxidase (HRP)-conjugated polyclonal secondary antibody for 2 h. After 3 washes as indicated above, signal detection was performed using a chemiluminescence kit (Perkin-Elmer).

### Western blotting

For DARPP-32 analysis in NAc, brains were rapidly removed and tissue from NAc dissected 15, 45, or 90 min following SKF 83959 administration, and were flash frozen until ready for use. For evaluation of pERK1/2 expression brains were removed immediately following place preference testing. Tissue was suspended in cell lysis buffer containing protease and phosphatase inhibitors (Thermo Scientific) and 30–50 μg of protein were incubated in sample buffer for 3 min at 95° C. Proteins were resolved by electrophoresis on 10% polyacrylamide gels under denaturing conditions (SDS–PAGE) and transferred onto nitrocellulose or PVDF membranes (Bio-Rad Laboratories, Hercules, CA) using a semidry transfer system (Invitrogen). Membranes were blocked in TBS-Tween (TBS-T)/5% nonfat milk for 1 h followed by incubation with PBS-T/5% nonfat milk containing the indicated first antibody overnight at 4°C. Membranes were washed in TBS-T (3 × 10 min) and incubated with the appropriate horseradish peroxidase (HRP)-conjugated polyclonal secondary antibody (Anti-rabbit or anti-mouse, BioRad) for 2 h at room temperature. After three washes as indicated above, signal detection was performed using a chemiluminescence kit (Perkin-Elmer). The primary antibodies used were: anti-phospho-Thr75-DARPP-32 (1:1,000), anti-phospho-Thr34-DARPP-32 (1:2,000), rabbit anti-phospho-ERK1/2 (Sigma, 1:1,000), rabbit anti-GAPDH (Abcam; 1:10,000).

### Surgery

Rats were anesthetized with isoflurane (5%), administered analgesic ketoprofen (5 mg/kg, s.c.) and secured in a stereotaxic frame. A cannula (22-gauge, Plastics One) was placed unilaterally into the intracerebroventricular space close to the midline according to the following stereotaxic coordinates: AP −0.8 mm, ML + 1.3mm, DV – 3.7mm, and was secured by dental cement anchored with four stainless steel screws (Plastics One) fixed on the dorsal surface of the skull. AP and ML coordinates were taken from bregma, DV coordinate from the dura (Paxinos and Watson, 1988).

Electrodes (diodes, Plastics One) for electrophysiology studies were placed unilaterally into NAc (AP: +1.6, L: −1.2, V: −7.2) with ground electrode over the cerebellum. The animals were allowed to recover in their home cage for a minimum of 5–10 days before the recording experiments were performed. Cannula and electrode placements were visually validated postmortem in brain slices. For the self-administration studies, a catheter constructed of silastic tubing was surgically implanted in the right jugular vein. The terminal end of the catheter consisted of a 22-gauge guide cannula (Plastics One) and was anchored subcutaneously between the scapulae with a small piece of Marlex mesh.

### Electrophysiology

Baseline LFP oscillatory recordings were taken from NAc of anesthetized animals for 5 min. Rats were then injected with SKF 83959 (0, 1.5 mg/kg, sc) followed 5 min later by cocaine (0, 10 mg/kg ip). Fifteen minutes following cocaine injection recordings were initiated for an additional 5 min. Date was collected in 1 min bins and sampled at a rate of 5,000 samples/second. Data was analyzed using routines from the Chronux software package (chronux.org) for MATLAB (MathWorks), downsampled at 1,000 Hz and segmented (5s window). Data was filtered (Butterworth) and detrended (mean subtracted and linear trend removed). Continuous process multitaper time-frequency spectral analyses were performed on baseline and drug-induced LFP signals. Multitaper spectral power was calculated for each segment in the following frequency bands: slow/delta (1.0–4 Hz); theta (4–8 Hz); alpha (8–13Hz); beta (13–30 Hz); and gamma (30–70 Hz). Data for each frequency band was averaged over segments and within groups using SPSS statistical package and changes in spectral power compared between drug-treated groups and controls.

### Behavioral procedures

#### Conditioned place preference

The place preference chamber (Harvard Apparatus, UK) consisted of two interconnected equally sized compartments (45 cm H × 34 cm W × 40 cm L). The compartments were differentiated by the wall motifs, color and floor texture.

The animals were first habituated to the CPP chambers for 2 days, followed by the measurement of their baseline preference for each of the two chambers. The baseline preference determined which side of the chamber the drug would be paired for a balanced experimental design. Cocaine was delivered in the least preferred chamber (defined from baseline preference). For SKF 83959 and since it was not know if this drug would lead to CPP or CPA half of animals received SKF 83959 in the preferred side whereas the other half received it in the non-preferred side. Habituated rats underwent 6 days of conditioning sessions, during which they received 3 drug treatments and 3 saline treatments in alternating order (D-S-D-S-D-S). The drug treatments were: vehicle, 1.0 mL/kg; SKF 83959, 1.0 mg/kg, s.c.; cocaine, 10 mg/kg, i.p.; TAT-D1 peptide, 300 pmol, i.c.v.; TAT-Sc peptide, 300 pmol, i.c.v., or a combination of these drugs. Immediately after the injection the rats were confined to the assigned chamber for 30 min. The TAT-D1 peptide was given 15 min prior to the start of each conditioning session. On the test day, the rats were allowed to freely explore the two chambers, and the time the animals spent in each chamber was recorded. To examine the effects of the D1-D2 heteromer on the expression of cocaine CPP, animals first underwent 6 days of conditioning sessions with cocaine (10 mg/kg, i.p.) and saline in alternating order. On the test day, the animals received an injection of SKF 83959 (2.5 mg/kg, s.c.) or vehicle, and the time they spent in each chamber was recorded. Place preference or aversion was established if the animal spent significantly more or less time in the drug-paired in comparison to the baseline preference, and thus each rat acted as its own control. Animals that spent more than 40% of total time in the middle compartment were excluded.

#### Locomotor sensitization

The animals were first habituated to the locomotor chamber (20 cm H × 25 cm W × 40 cm L equipped with two arrays of 16 infrared photocells and automated recording of horizontal locomotor activity) for 3 days following which they received the assigned drug treatment daily for 7 days (saline, 1.0 ml/kg, i.p.; SKF 83959, 0.4 mg/kg, s.c.; TAT-D1 peptide, 300 pmol, i.c.v.; cocaine, 10 mg/kg, i.p.; cocaine + SKF 83959, cocaine + TAT-D1 peptide). The dose of SKF 83959 used was previously shown to attenuate amphetamine-induced locomotor sensitization without desensitizing the D1-D2 heteromer over repeated injections (Shen et al., 2014), while the dose of cocaine was the same as described by Robison et al. (2013). The locomotor activity of the animals was measured for a total of 60 min daily, 30 min prior and 30 min following the assigned treatment over 7 injection days. All animals received a single injection of priming cocaine (5.0 mg/kg, i.p.) on day 8 following a 24 h withdrawal, and their locomotor activity was again evaluated.

#### Intravenous self-administration

Adult male Sprague-Dawley rats under a restricted diet were first trained to lever-press for food under a FR1 schedule. Rats were allowed a total of 100 food pellets during each 30 min training session. The rats that consumed 100 pellets for 3 consecutive days were considered lever-trained. Trained animals then underwent surgery for jugular vein intravenous catheter implantation to allow for IV cocaine infusion. The animals were allowed to recover from surgery for a week, and then were first trained for cocaine SA under the FR1, FR3, and then eventually FR5 schedules of reinforcement. Cocaine infusions were delivered only when the left (active) lever was pressed, and the delivery was associated with a 5 s light cue located directly above the left lever. Pressing of the right (inactive) lever had no functional consequence. For each infusion the animals received 0.25 mg cocaine/0.1mL/5.5s. Once stable responding was achieved, the dose response relationship of D1-D2 heteromer stimulation (SKF 83959, 0, 0.5, 1.5 mg/kg, s.c.) and cocaine SA under the FR5 schedule was examined. Each animal received every dose of SKF 83959 (0; 0.5; 1.5 mg/kg SKF 83959) or vehicle (0 mg/kg SKF 83959), randomized, 48 h apart. Animals who received SKF 83959 still were able to press a “normal” amount of presses when they received vehicle 48 h later. The number of inactive lever presses was unchanged. Next, the animals underwent extinction training, during which no cocaine was infused following active lever presses. Extinction was achieved when responding on the active lever had reached a stable level of less than 15 responses over 2 h. Once the extinction training was completed, the animals underwent surgery for intracerebroventricular cannula implantation for TAT-D1 peptide infusions. Following a week of recovery, the effects of D1-D2 heteromer stimulation (SKF 83959, 0.5, 1.5mg/kg, s.c.) and inactivation (TAT-D1 peptide, 300 pmol, i.c.v.) on cocaine- (5 or 10 mg/kg, i.p.) induced reinstatement of SA was then investigated, with cue-induced reinstatement assessed 1 week later. For both the cocaine and cue reinstatement experiments, each animal underwent each treatment, randomized, 48 h apart.

#### Sucrose preference test

Sucrose preference in a two-bottle sucrose-water choice test was assessed in untrained animals. Sucrose solution (1%, w/v) was available for 2 h and sucrose consumption was measured. Water was removed from the rat cages for a period of 2 h prior to the test. Two plastic bottles with the normal water bottle stoppers containing either water or 1% sucrose were then placed on the cages and, immediately following SKF 83959 (2.5 mg/kg, s.c.) or TAT-D1 peptide (300 pmoles/4μl, i.c.v.) administration, animals were allowed to freely consume fluids for 2 h.

##### Statistical analysis

Results are reported as mean ± SEM. Immunoblot data was collected by densitometry and expressed as a percent of controls. Following the appropriate ANOVA, comparisons were performed using Bonferroni *post-hoc* tests. For the locomotor sensitization data the dependent measure was horizontal activity (beam breaks). For CPP data, the statistical significance of each dependent measure was first evaluated using an ANOVA with Treatment as the between-subjects factor followed by Student's paired *t*-tests for *post-hoc* comparisons. For the dose response study that examined the effect of SKF 83959 on the maintenance of cocaine SA under the FR5 schedule of reinforcement, the active lever response was analyzed using repeated measures of ANOVA with Dose (0, 0.05, 0.5, 1.5 mg/kg SKF 83959) as the within-subject factor. For the reinstatement studies, the active lever response was analyzed using repeated measures of ANOVA with Treatment (Cocaine, Cue, SKF 83959, TAT-D1 peptide, Cocaine+SKF 83959, Cue+SKF 83959, Cocaine+TAT-D1 peptide) as the within-subject factor. Planned comparisons between groups were done using paired Student's *t*-test.

Computations were performed using the SPSS/PC+ statistical package.

## Author contributions

SG and AH: Designed the project; SG, AH, MS, MP, PF, BO: Designed the experiments; AH, MP, MS, TF, TN, MA: Performed experiments; SG, AH, MP, MS, TB, AG, PF: Analyzed the data; SG and AH: Wrote the manuscript. We thank Dr. Marcos Sanches for his guidance and review of the statistical methods used.

### Conflict of interest statement

AG reports that while he has no direct conflicts of interest, he is or was a consultant with the following: Johnson&Johnson, Lundbeck, Pfizer, GSK, Merck, Takeda, Dainippon Sumitomo, Otsuka, Lilly, Roche, Asubio, Abbott, Autofony, Janssen, Alkermes. The other authors declare that the research was conducted in the absence of any commercial or financial relationships that could be construed as a potential conflict of interest. The handling editor declared a past co-authorship with one of the authors SG.

## Supplementary material

Supplementary Figure 1 PLA controls.

Supplementary Figure 2 Specificity of PLA: use of TAT-D1 peptide.

Supplementary Figure 3 Z-stack PLA signal between D1R and D2R in rat NAc.

Supplementary Figure 4 PLA signal between D1R and D2R in monkey NAc.

Supplementary Figure 5 Colocalization of dopamine D1R and D2R using Immunohistochemistry in rat NAc.

Supplementary Figure 6 TAT-D1 peptide on D1-D3 and D2-5HT2A heteromers.

Supplementary Figure 7 **(A)** Validation of intra-NAc injections. **(B)** Modulation of ΔFOSB.

Supplementary Figure 8 Cell specific effects of D1-D2 heteromer activation on phospho-Thr75-DARPP32.

Supplementary Video 1 A short movie: Z-stacks showing D1-D2 heteromers through PLA.
