# Supplementary material for: Activation of Dopamine D1-D2 Receptor Complex Attenuates Cocaine Reward and Reinstatement of Cocaine-Seeking through Inhibition of DARPP-32, ERK, and ΔFosB
Source: Front Pharmacol. 2018 Jan 4;8:924. doi: 10.3389/fphar.2017.00924 (PMC5758537; doi:10.3389/fphar.2017.00924)
Supplement: Supplementary Figure 1 — PLA controls. [file Presentation1.PDF]

Supplementary Fig. 1: PLA Controls

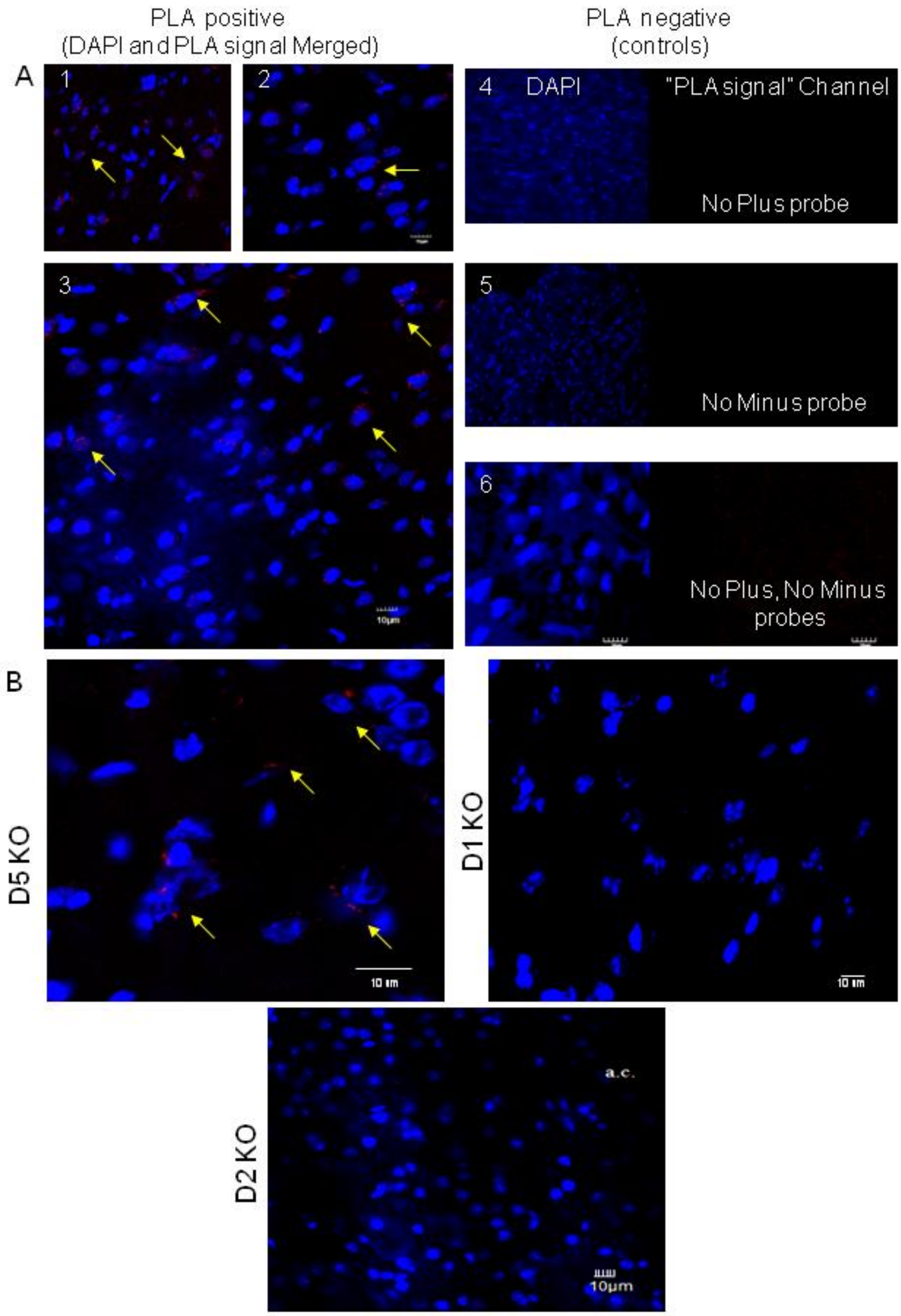

Legend for Supplementary Fig. 1. PLA Controls

Positive PLA signal is shown in the left side of Supplementary Fig. 1A. No PLA signal was observed in the absence of one or the two probes (Supplementary Fig. 1A, right). The PLA signal was observed in the dorsal and ventral striatum of D5 KO mouse, whereas it was absent in D1 KO and D2 KO mouse striatum, a clear indication of the specificity of the probes (Supplementary Fig. 1B).

Supplementary Fig. 2. Specificity of PLA: Use of TAT-D1 peptide

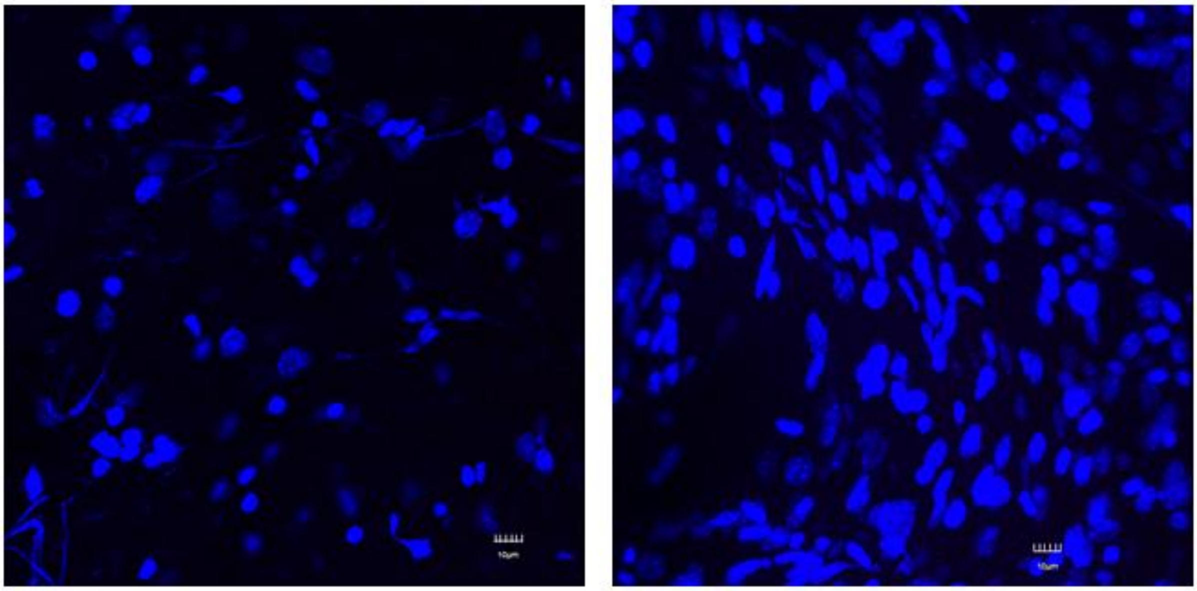

Legend: Specificity of PLA: Use of TAT-D1 peptide

Pretreatment with the TAT-D1 peptide (300 pmol, i.c.v.) abolished the PLA signal in rat striatum as shown by a 2D image (left) and a Z-stack generated 3D image (right).

Supplementary Fig. 3
Z-stack PLA signal between D1R and D2R in rat NAc

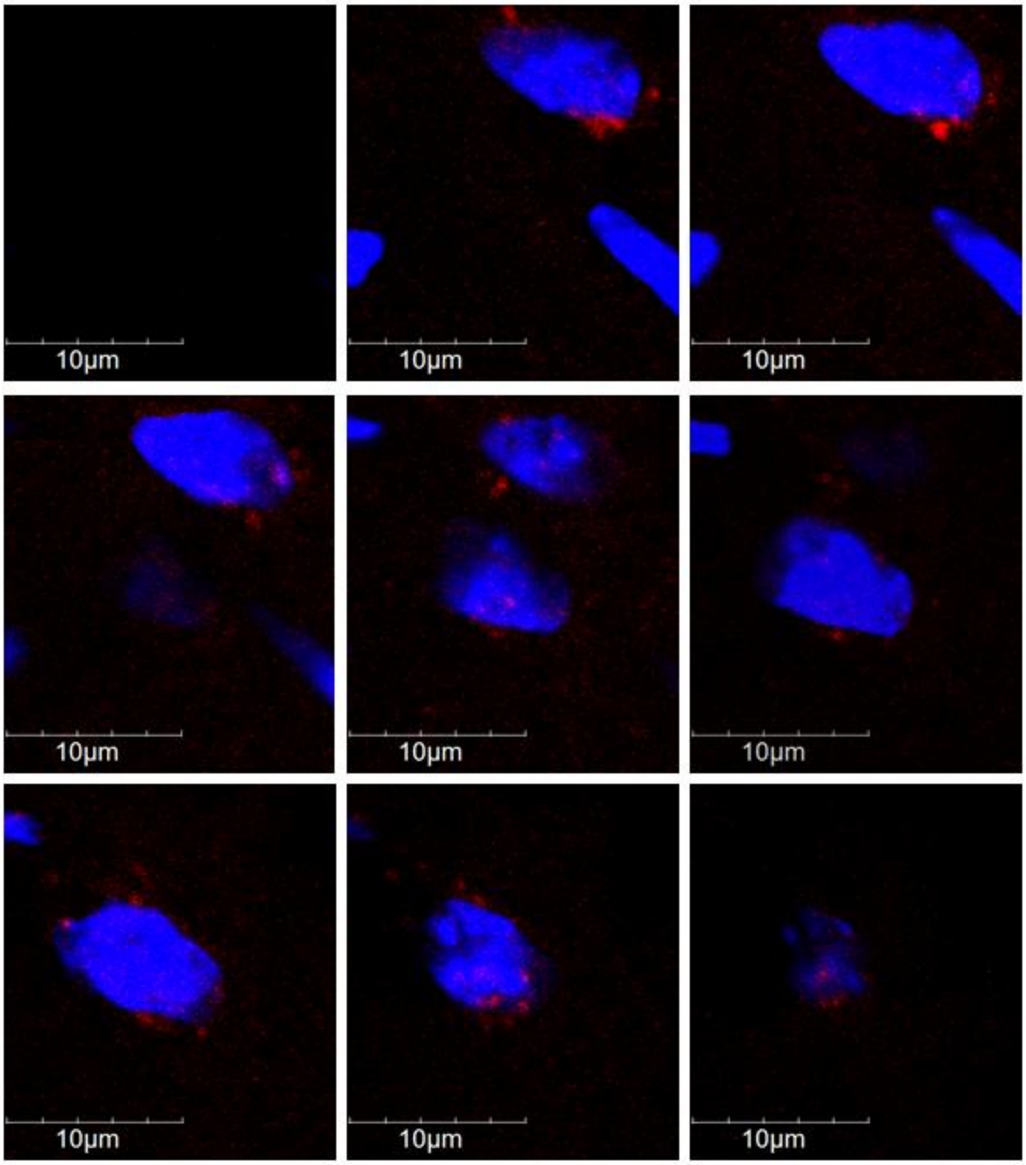

Legend: Z-stack PLA signal between D1R and D2R in rat NAc

Z-stacks were taken and their analysis showed that the PLA signal was around neuronal cell bodies, suggesting that D1-D2 heteromers were at the cell surface (Supplementary Fig. 3 and Z-stacks video)

Supplementary Fig. 4
PLA signal between D1R and D2R in monkey NAc

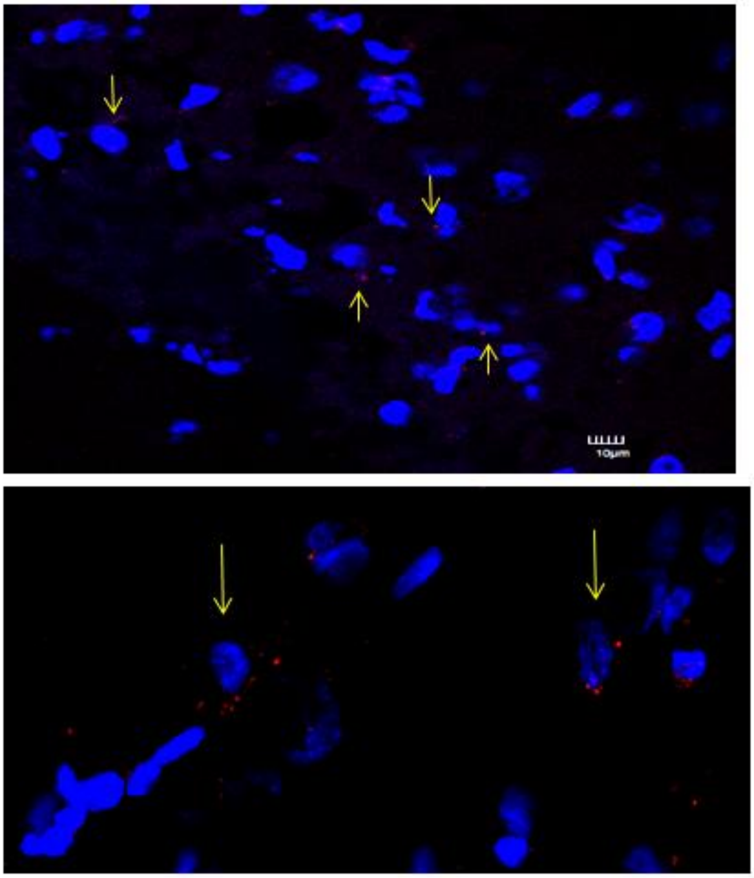

Legend: PLA signal between D1R and D2R in monkey NAc

The PLA signal was also detected in monkey NAc and caudate, indicative of a close proximity between dopamine D1 and D2 receptors in non-human primate.

Supplementary Fig. 5

Colocalization of dopamine D1R and D2R using Immunohistochemistry in rat NAc

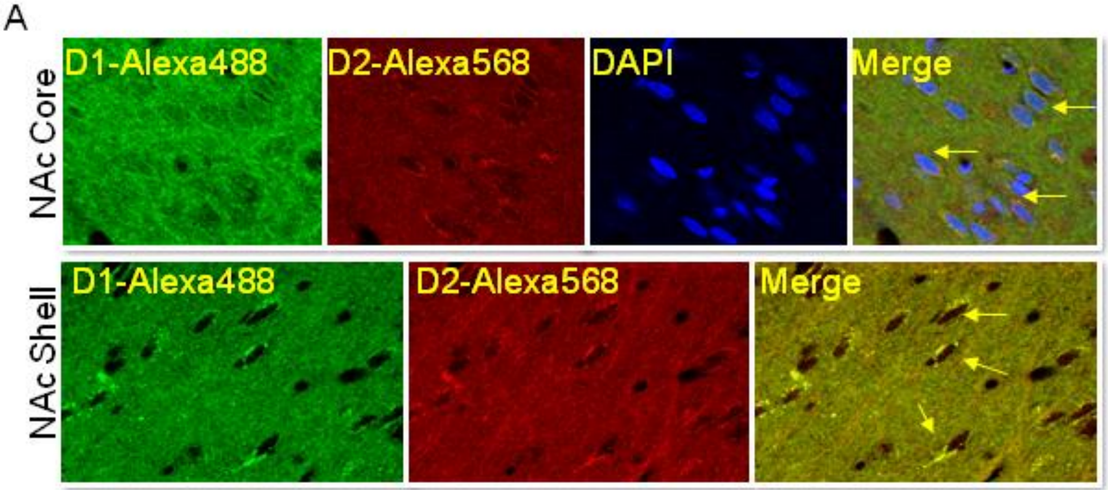

Legend: Immunohistochemistry of D1R and D2R in rat NAc

The primary antibodies for D1R and D2R were directly conjugated to Alexa-488 and Alexa-568, respectively, avoiding the use of secondary antibodies. The IHC results (Fig. 1B and Supplementary Fig. 5) confirmed that D1R and D2R colocalized in at least 20% of D1R-expressing neurons in the NAc-core and in more than 25% of D1R-expressing neurons in the NAc-shell.

Supplementary Figure 6

BRET experiments with D1-Rluc or D2-Rluc as donors
and D3-GFP or 5HT2A-GFP as acceptors.
BRET saturation curves showed that D1-D3 and D2-5HT2A formed heteromers.
Pre-treatment with TAT-D1 peptide had no effect on both heteromers.

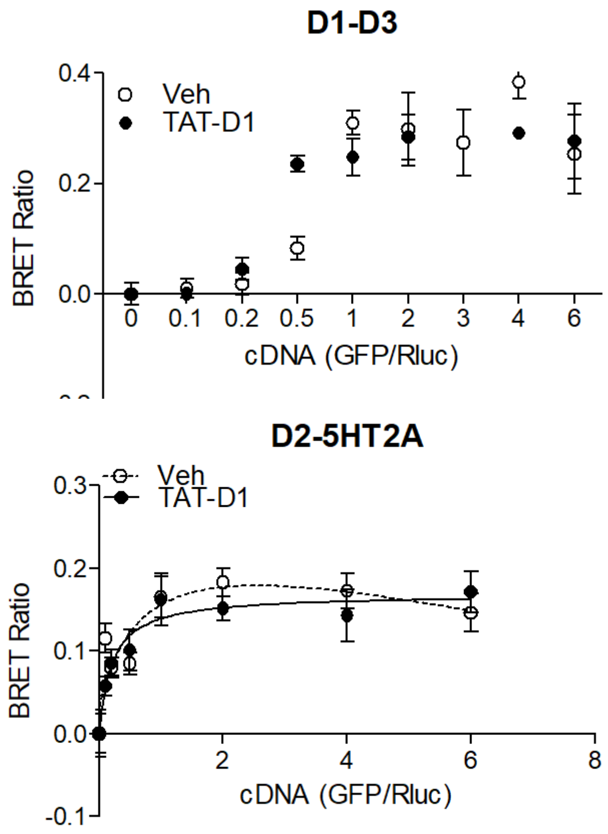

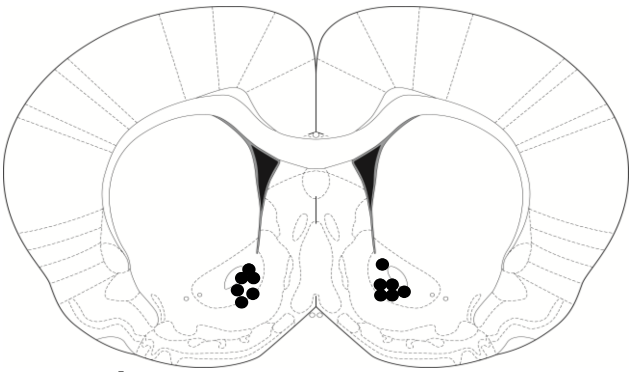

Supplementary Fig. 7A **Intra-NAc injection sites of roscovitine**

The sites of injection were visually verified.

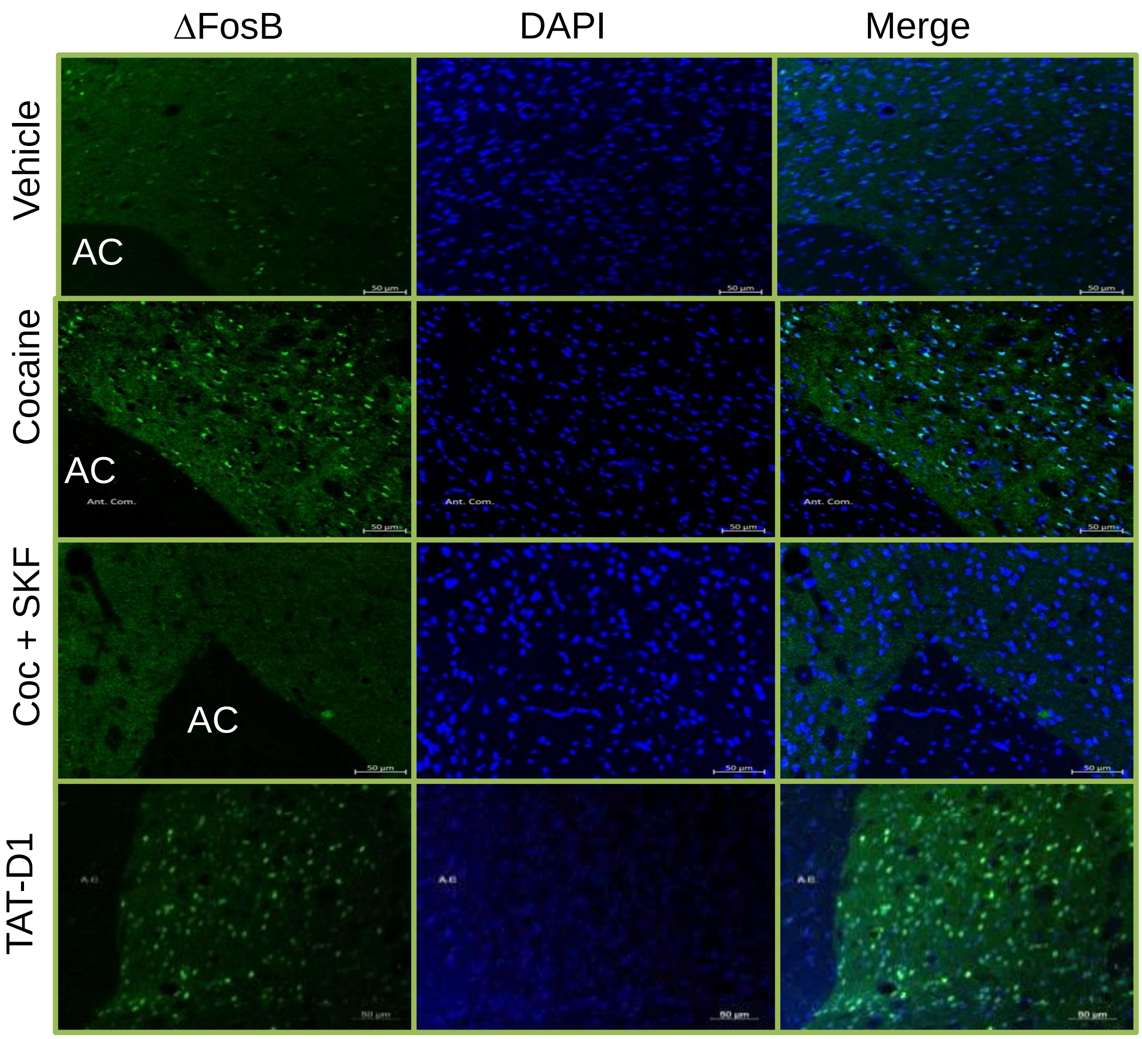

Supplementary Fig. 7B **Modulation by ΔFosB.**

Chronic cocaine (7 injections) induced an increase in ΔFosB, which was blocked by co-injection with SKF 83959. Chronic disruptiion of the D1-D2 heteromer by TAT-D1 (7 injections) had the same effect as cocaine (increased ΔFosB). The image shown is from NAc-core, but the effects were observed in CPu and NAc-shell as well.

Supplementary Figure 8

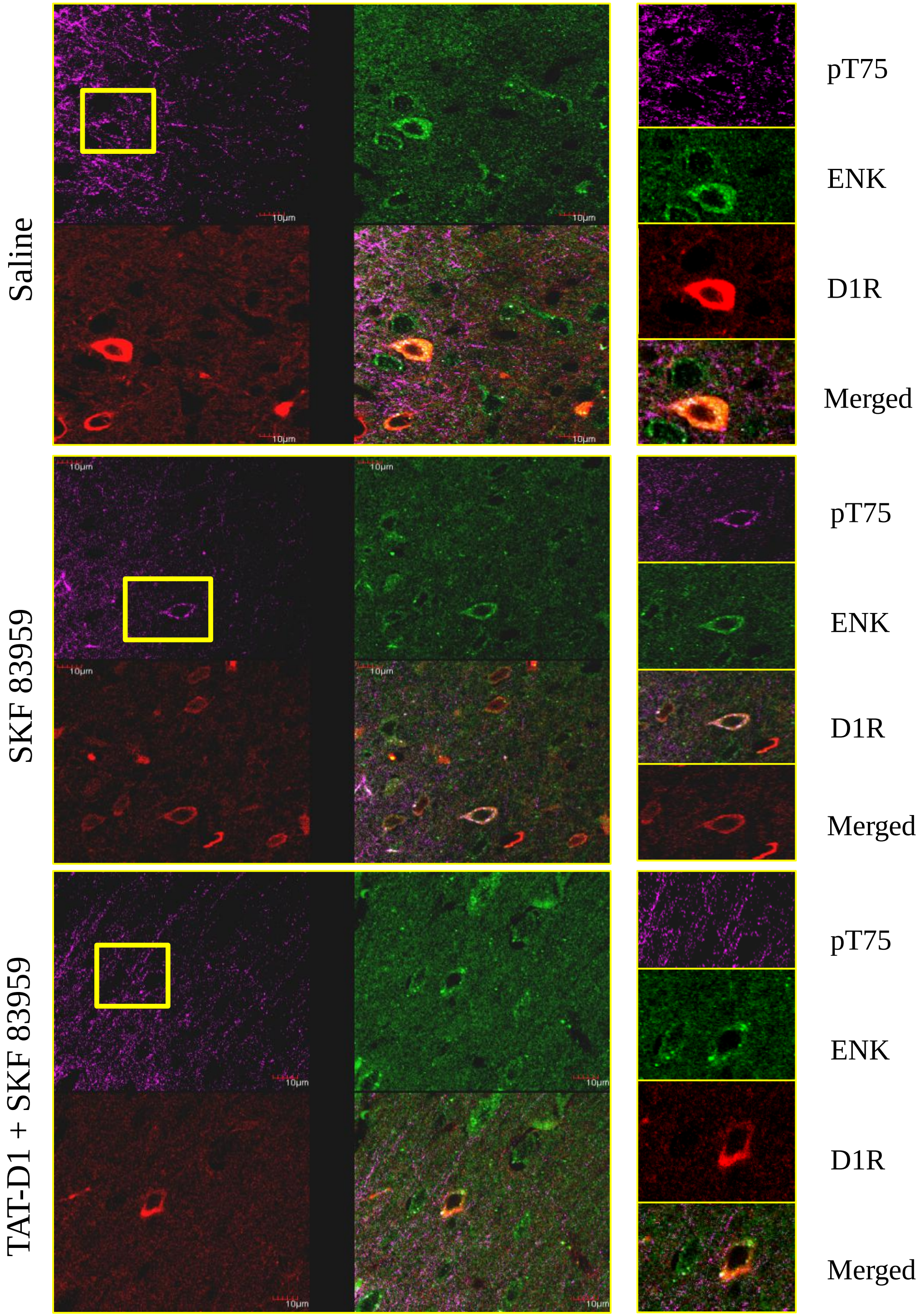

Effect of D1-D2 heteromer on phospho-Thr75-DARPP 32 (pT75).
SKF 83959 increased pT75 exclusively in D1R –ENK (D1-D2)-expressing neurons.
Pre-treatment with TAT-D1 peptide blocked this effect suggesting that the SKF 83959-induced increase in pT75 was mediated through D1-D2 heteromer. D1R (red), ENK (green), pT75 (magenta).
